# Role of Ni in PtNi Bimetallic Electrocatalysts for Hydrogen and Value-Added Chemicals Coproduction via Glycerol Electrooxidation

**DOI:** 10.1021/acscatal.2c03907

**Published:** 2022-11-10

**Authors:** Hui Luo, Victor Y. Yukuhiro, Pablo S. Fernández, Jingyu Feng, Paul Thompson, Reshma R. Rao, Rongsheng Cai, Silvia Favero, Sarah J. Haigh, James R. Durrant, Ifan E. L. Stephens, Maria-Magdalena Titirici

**Affiliations:** † Department of Chemical Engineering, Imperial College London, South Kensington Campus, LondonSW7 2AZ, U.K.; ‡ Chemistry Institute and Center for Innovation on New Energies, State University of Campinas, P.O. Box 6154, São Paulo13083-970, Campinas, Brazil; § School of Engineering and Materials Science, Queen Mary University of London, LondonE1 4NS, U.K.; ∥ XMaS CRG, ESRF, 71 Avenue des Martyrs, Grenoble38000, France; ⊥ Department of Materials, Imperial College London, South Kensington Campus, LondonSW7 2AZ, U.K.; # School of Materials, University of Manchester, Oxford Road, ManchesterM13 9PL, U.K.; ¶ Centre for Processable Electronics, Imperial College London, LondonSW7 2AZ, U.K.; ∇ Department of Chemistry, Imperial College London, South Kensington Campus, LondonSW7 2AZ, U.K.; ○ Advanced Institute for Materials Research (WPI-AIMR), Tohoku University, 2-1-1 Katahira, Aobaku, Sendai, Miyagi980-8577, Japan

**Keywords:** glycerol oxidation, electrocatalysis, PtNi nanoparticles, glycerol adsorption, operando spectroscopy, product distribution

## Abstract

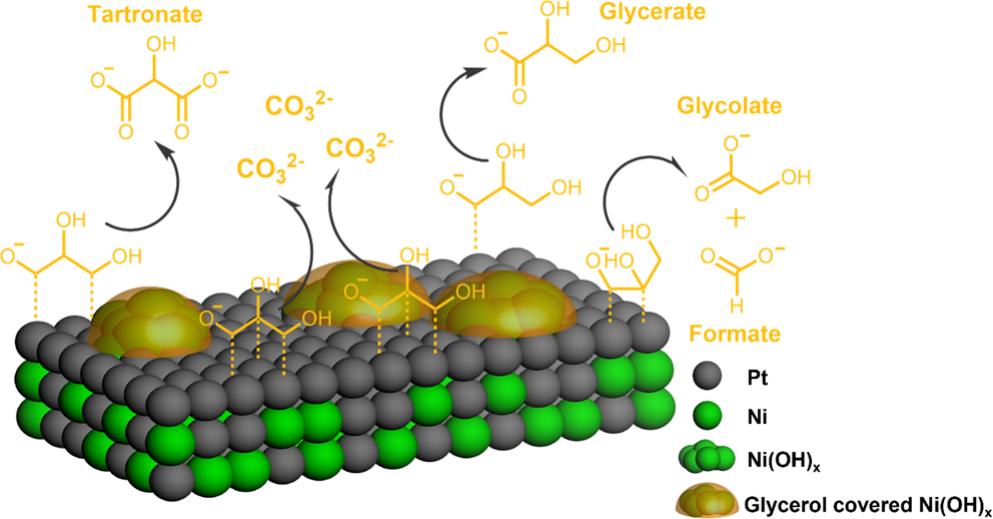

Pt-based bimetallic electrocatalysts are promising candidates to convert surplus glycerol from the biodiesel industry to value-added chemicals and coproduce hydrogen. It is expected that the nature and content of the elements in the bimetallic catalyst can not only affect the reaction kinetics but also influence the product selectivity, providing a way to increase the yield of the desired products. Hence, in this work, we investigate the electrochemical oxidation of glycerol on a series of PtNi nanoparticles with increasing Ni content using a combination of physicochemical structural analysis, electrochemical measurements, operando spectroscopic techniques, and advanced product characterizations. With a moderate Ni content and a homogenously alloyed bimetallic Pt–Ni structure, the PtNi2 catalyst displayed the highest reaction activity among all materials studied in this work. In situ FTIR data show that PtNi2 can activate the glycerol molecule at a more negative potential (0.4 *V*_RHE_) than the other PtNi catalysts. In addition, its surface can effectively catalyze the complete C–C bond cleavage, resulting in lower CO poisoning and higher stability. Operando X-ray absorption spectroscopy and UV–vis spectroscopy suggest that glycerol adsorbs strongly onto surface Ni(OH)_*x*_ sites, preventing their oxidation and activation of oxygen or hydroxyl from water. As such, we propose that the role of Ni in PtNi toward glycerol oxidation is to tailor the electronic structure of the pure Pt sites rather than a bifunctional mechanism. Our experiments provide guidance for the development of bimetallic catalysts toward highly efficient, selective, and stable glycerol oxidation reactions.

## Introduction

The glycerol electrooxidation reaction (GEOR) to coproduce green H_2_ and valuable chemicals at low potential constitutes a promising strategy to phase out fossil fuels in the H_2_ and chemical sector. Each year, a large surplus of glycerol is produced (7.66 Mt) compared to its demand (3 Mt), consequently reducing the price of glycerol to $0.11 kg^–1^,^1^ making it a suitable and low-cost feedstock for H_2_ production. As the market for glycerol derivatives, such as glyceric acid, tartronic acid, and lactic acid, has grown significantly, this approach would lead to an overall better economic and environmental impact. As a result, the GEOR reaction has been extensively studied during the last decades.^2−4^

**Figure 1:**
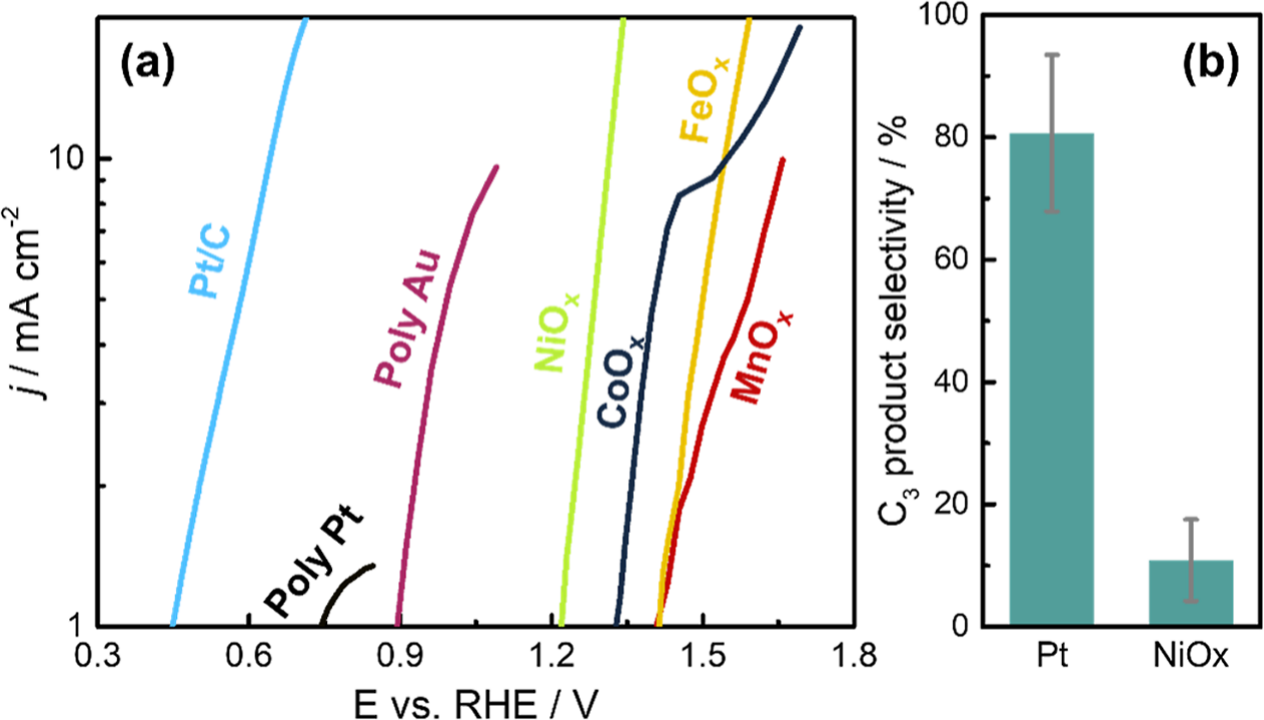
Overview of the functionality of different electrocatalysts for GEOR from the literature and from the present work. (a) Current density in alkaline media. Data adapted from: ref (5) for polycrystalline Pt (Poly Pt) and Au (Poly Au); ref (6) for NiO_*x*_, FeO_*x*_, CoO_*x*_, and MnO_*x*_. Data for Pt/C are from the present study. (b) Faradaic selectivity toward C_3_ products. Data for Pt were averaged from refs (5), (7), and (8) at a potential between 0.5 and 0.9 *V*_RHE_; data for NiO_*x*_ were averaged from refs (6) and (9) at a potential above 1.3 *V*_RHE_.

In general, a good GEOR catalyst should be able to activate the glycerol molecule at a relatively negative potential and have fast kinetics to reach high current density. At the same time, it should also exhibit high selectivity toward desired anodic chemical products, that is, C_3_ chemicals. Pt-based catalysts are currently the most studied in the literature owing to their high activity at more negative potentials (Figure 1a,b) and the ability to break various chemical bonds.^10,11^ However, the side effect of this high bond-breaking ability is that pure Pt materials can suffer from limited reactivity, poor product selectivity, and severe poisoning from the generated CO and other intermediates.^12−16^

In recent studies, attention has been paid to using Pt–Ni bimetallic electrocatalysts for glycerol oxidation due to Ni’s abundant earth reserve and high oxidation activity. This combination can significantly improve the GEOR and similar CO or alcohol oxidation activity and reduce the amount of costly Pt needed.^17−25^ However, despite the high performances reported in the literature,^26−29^ very few in-depth studies on the exact role of Ni were performed. This is due to the lack of information on how the Ni species interact with glycerol. When it comes to mechanistic interpretation, studies often refer to the previous investigations on Pt–Ru for CO/methanol oxidation, where two main contributions were proposed: (i) the “bifunctional mechanism,” where the binding to *C and *O species is independent of each other on two separate surface sites, hence activating each species separately: Pt*CO + Ni*OH →Pt* + Ni* + CO_2_ + H^+^ + e^–^, where * denotes a vacant metal site at the surface and *OH and *CO are surface adsorbed hydroxide and CO;^30−37^ (ii) the electronic effect, where the electronic structure of a pure Pt surface is changed when some Pt atoms are substituted by Ni, which will weaken the Pt*–CO and other Pt*–C_*n*_ bond interactions, allowing the carbon species to be more easily oxidized directly by *OH adsorbed on the Pt surface. The Ni substation can also increase water activation and the Pt*–OH bond strength, thereby allowing the reaction of OH and CO directly on the Pt.^38−41^ For CO and methanol oxidation, both mechanisms may exist, but the dominant mechanism depends critically on the Ru island size and dispersion over the Pt surface. Ramaker and co-workers found that monodispersed Ru islands promote the bifunctional mechanism, while the electronic effect dominates in the presence of larger Ru islands.^22,42^ Although it is not yet clear whether the findings on bimetallic Pt–Ru catalysts can be translated to Pt–Ni in GEOR, this knowledge provides guidance for studying the role of Ni in Pt–Ni bimetallic electrocatalysts. Similar to Pt–Ru, Pt–Ni bimetallic catalysts studied in alkaline environment will also form Ni(OH)_*x*_ islands on the Pt surface. This is because the alkaline condition precludes the dissolution of either Pt or Ni, causing Ni(OH)_*x*_ formation and enrichment.^36^

The investigation of pure Ni-based oxides for GEOR is thus necessary. It has been reported that they can only catalyze the reaction at potentials more positive than 1.3 *V*_RHE_, far more positive than Pt (see Figure 1a); the main product formed under such conditions is formic acid_._^6,43−45^ Fleischmann et al.^46^ observed that the oxidation of glycerol coincides with the onset of the redox wave in the absence of glycerol. In this context, we could conjecture that reactive species such as *O or *OH formed during the oxidation of Ni are the reactive species for glycerol oxidation, similar to the bifunctional mechanism on Pt surfaces. However, the fundamental reason why Ni(OH)_*x*_ requires such high potential is unclear.

Besides activity, we anticipate that the composition of the PtNi bimetallic catalyst can also influence product selectivity, which is largely determined by three factors: (i) the adsorption energy of glycerol and the more oxidized intermediates;^47^ (ii) the adsorption energy of *OH; (iii) the energy barriers between the intermediate steps which are higher for C–O and C–C breaking than for the dehydrogenation steps.^48,49^ For example, Wieckowski and co-workers found that C–C cleavage becomes increasingly more exothermic when the degree of dehydrogenation of ethanol increases.^49^ While factors (i) and (ii) would be controlled by purely electronic effects, the barriers may also be controlled by other factors, such as the geometry of the surface sites^50^ and pH.^51^ In this case, the Ni content in PtNi may also have an effect on the glycerol oxidation selectivity.

Herein, we present a systematic study of glycerol oxidation on three different PtNi bimetallic nanoparticle electrocatalysts with different Pt to Ni ratios. A simple and effective solvothermal route to produce PtNi bimetallic nanoparticle electrocatalysts with tunable compositions was adapted from Strasser and co-workers.^52^ By increasing the amount of Ni precursor content, three different PtNi bimetallic nanoparticles are formed, denoted as PtNi1, PtNi2, and PtNi3, which display distinctive catalytic performance for GEOR. Extensive physicochemical and electrochemical characterizations were employed to gain a full picture of the pristine structure of these catalysts. We discovered that, among all three catalysts, PtNi2 exhibits the highest glycerol oxidation activity and the lowest poisoning rate. Using a combination of electrochemical stripping and operando spectroscopic techniques, we show direct evidence of strong glycerol adsorption onto surface Ni species, which provides fundamental insights into the mechanism governing the enhanced activity in PtNi electrocatalysts. The Ni content and the homogenously alloyed structure within the PtNi catalysts play a crucial role in tuning the C–C cleavage ability: higher Ni content may facilitate partial C–C bond cleavage to glycolate and formate, whereas the more equally distributed Pt–Ni in PtNi2 can drive the complete glycerol oxidation to carbonates.

## Results and Discussion

**Figure 2:**
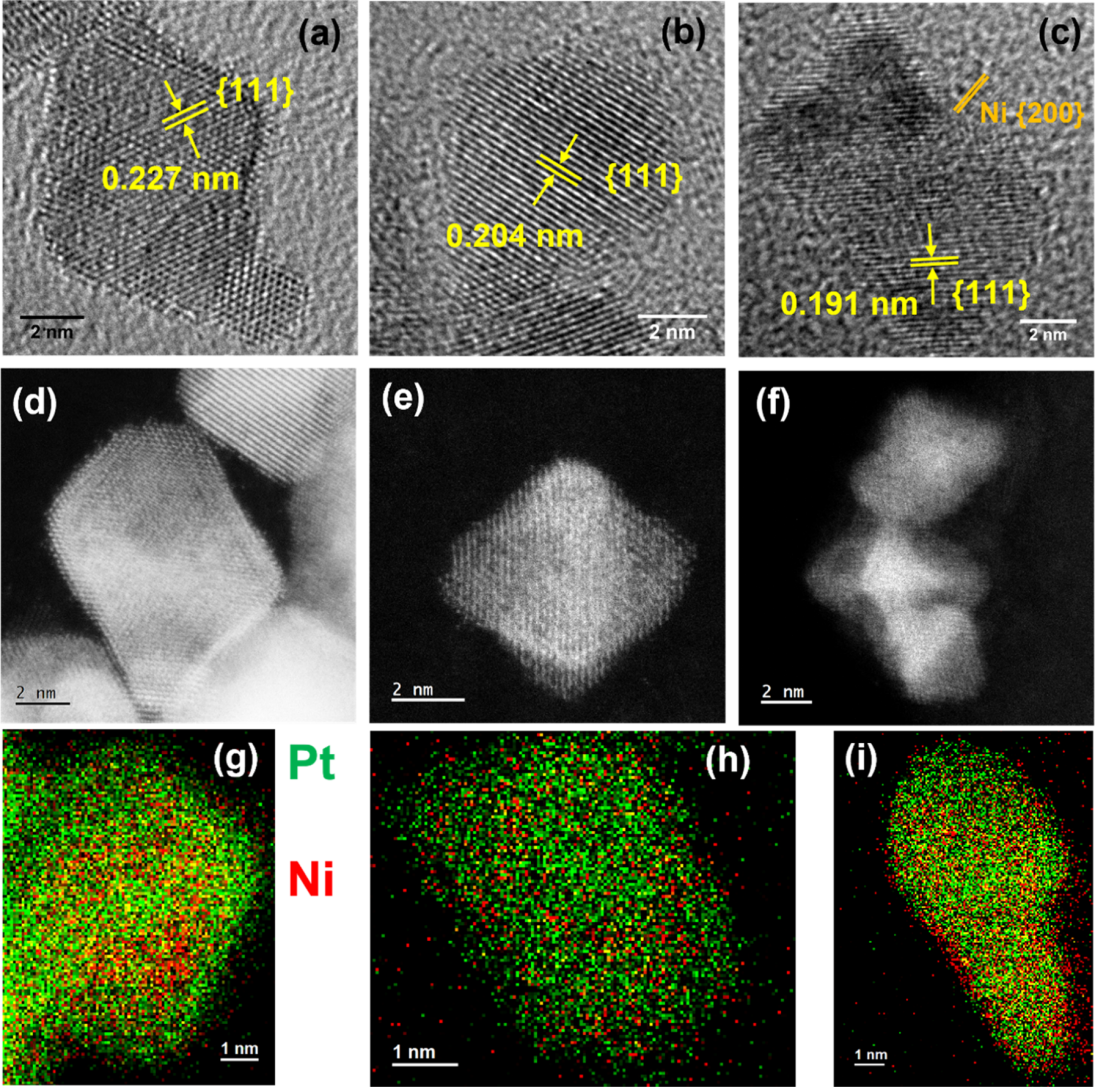
TEM bright field images and atomic-resolution aberration-corrected HAADF-STEM images of as-prepared catalysts PtNi1 (a,d), PtNi2 (b,e), and PtNi3 (c,f). (g–i). Selected STEM EDX mapping area on PtNi1 HAADF-STEM images in Figure S3: (g) PtNi1, (h) PtNi2, and (i) PtNi3. (green: Pt, red: Ni).

The morphologies of the as-prepared PtNi nanoparticles were first investigated with transmission electron microscopy (TEM). As shown in Figure 2a–c, the nanoparticles have a face-centered cubic (fcc) structure and a diameter of 5–8 nm. The particles adopt octahedral or cubic shapes dominated by {111} crystal facets with d-spacings for the {111} crystal planes (marked by pairs of yellow lines) measured to be 0.227, 0.204, and 0.191 nm for PtNi1, PtNi2, and PtNi3, respectively. Compared to the typical interplanar spacing for pure Pt {111} planes (0.231 nm), the shortened d-spacing in these nanoparticles indicates a bimetallic alloy structure for PtNi according to Vegard’s law.^53^ These observations are in line with the results obtained from X-ray diffraction (XRD). As shown in Figure S1, all samples display three main peaks indexed to (111), (200), and (220) planes of the fcc structure. Compared to standard diffraction patterns for the Pt (PCPDFWIN #70–2431) and Ni (PCPDFWIN #040–850) phases, the peaks for bimetallic PtNi nanoparticles show an obvious right shift resulting from the incorporation of Ni atoms into the Pt lattice.^54−56^ Although a few Ni nanoparticles can be found in the PtNi3 TEM images due to the excess amount of Ni precursor added, no diffraction peaks associated with this Ni monocomponent can be observed in XRD, suggesting its low concentration in the PtNi3 samples.

High-angle annular dark-field scanning transmission electron microscopy (HAADF-STEM) was used to investigate the distribution and atomic arrangement of Pt and Ni atoms in different individual PtNi electrocatalysts. The morphology and corresponding energy-dispersive X-ray spectroscopy (EDX)/electron energy loss spectroscopy mapping in Figures 2d–i and S2, S3 demonstrate that by increasing the Ni content, the obtained PtNi nanoparticles change from a Pt-rich surface in PtNi1 to a uniformly distributed Pt–Ni atomic structure in PtNi2, until a partially Ni-rich surface in PtNi3. X-ray photoelectron spectroscopy (XPS) was employed to investigate the surface composition of the as-synthesized samples. XPS exhibits an overall information depth of about 2.5 nm, with two-third of the signal stemming from the first 1 nm depth.^57^ XPS analysis shows that the Pt/Ni atomic ratios on the surface of PtNi samples are 2.2, 1.7, and 1.2 in PtNi1, PtNi2, and PtNi3, respectively, indicating that the external surface of these nanostructures is enriched with Pt. The total metal loading quantified by inductively coupled plasma-mass spectrometry (ICP–MS) shows a relatively stable Pt content among all catalysts, with increasing Ni percentage from PtNi1 to PtNi3 (Table S1 and Figure S4).

**Figure 3:**
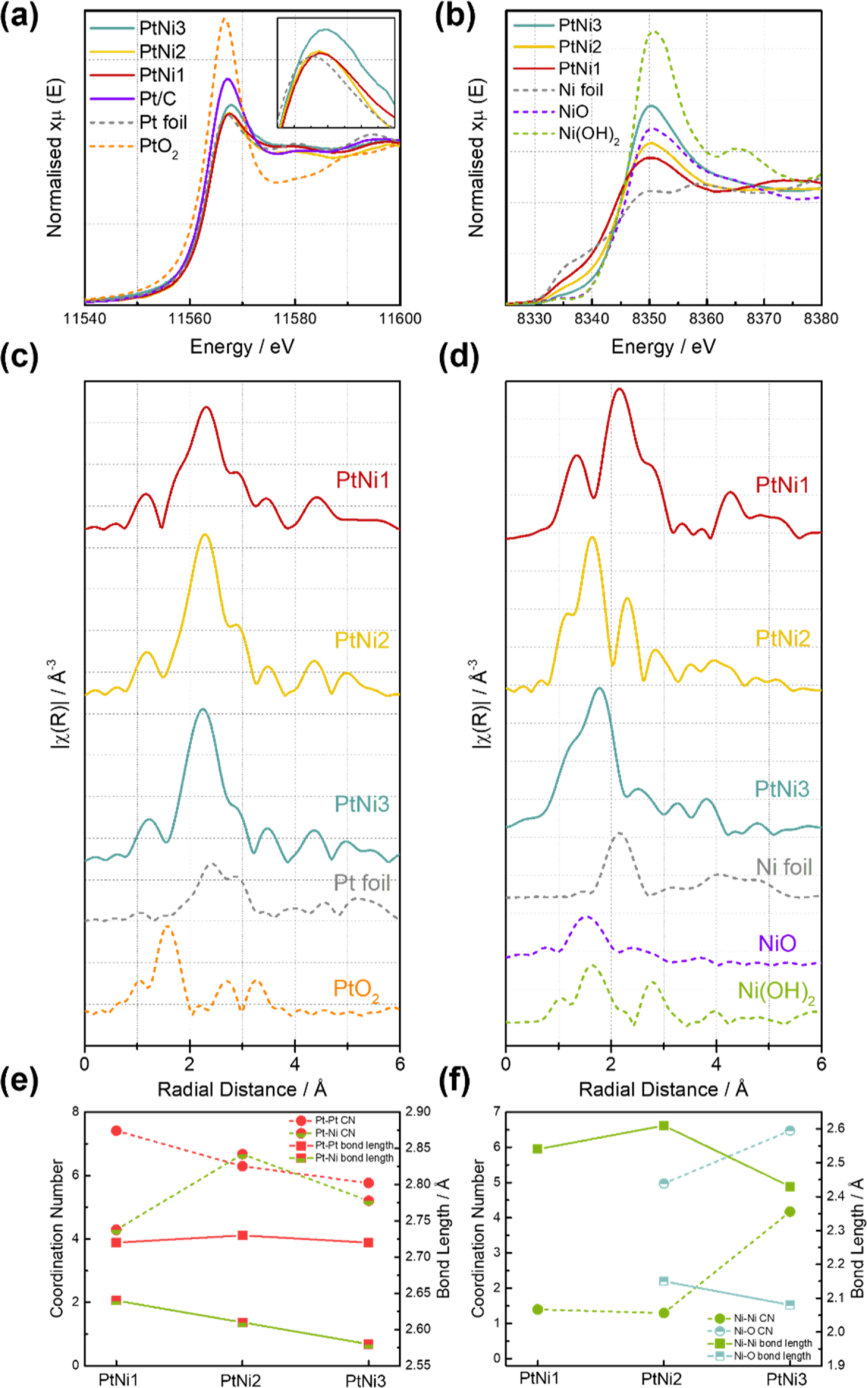
(a) Pt L_3_-edge (insert is an enlargement of the white line region) and (b) Ni K-edge XANES spectra of three as-prepared PtNi samples, ex situ, compared with reference materials; (c) Pt and (d) Ni EXAFS spectra showing the main bond formation in PtNi samples. The fitted results and fitting parameters are presented in Figures S6–S8 and Table S2. Local coordination number and bond length of (e) Pt–Pt and Pt–Ni and (f) Ni–Ni and Ni–O according to the fitted results in Table S2.

The local atomistic and electronic structure of Pt and Ni in the as-prepared PtNi materials was investigated with ex situ X-ray absorption spectroscopy (XAS) measurements. Figure 3a shows the X-ray absorption near edge structure (XANES) spectra at the Pt L_3_-edge. The overall XANES shapes for the bimetallic PtNi materials are similar to those of the Pt foil and Pt/C except for the edge absorption white line intensity. The white line magnitudes are smaller in the bimetallic PtNi catalysts than that in Pt/C, suggesting that Pt is less oxidized in the bimetallic structure.^58^ The Pt extended X-ray absorption fine structure (EXAFS) fitting results are included in Figures 3c, S5–S7, and Table S2. The Pt–Pt pair coordination number follows a descending trend from PtNi1 to PtNi3, indicating less Pt rich local areas, while the Pt–Ni pair coordination peak is the highest for PtNi2, which suggests that the atoms in this sample are most homogeneously coordinated, consistent with the STEM studies described above.

XANES for the Ni K-edge provides information on the oxidation state of Ni species.^59^ Most of the spectral features of PtNi catalysts (Figure 3b) are similar to those of previously reported PtNi alloy nanoparticles and partially match with NiO.^60,61^ Compared to the rather similar features of the Pt L_3_-edge in the three PtNi catalysts, the Ni K-edge XANES spectra showed more distinct differences. From PtNi 1 to 3, an increase in Ni oxidation states was observed. The Ni EXAFS fitting results indicate that more Ni–O bonds exist with increasing Ni contents from PtNi 1 to 3 (Figure 3d,f). This is in line with the increase in the surface Ni/Pt atomic ratio determined by XPS as Ni atoms at the surface are more prone to be oxidized in contact with the atmosphere.

Overall, the presented structural studies provide a clear picture of the atomic-scale distribution of the local chemical environment and atomic coordination in pristine PtNi bimetallic nanoparticles. The Pt–Pt atomic distance in all PtNi catalysts (2.72 Å, Figure 3e) remains constant, yet shorter than that in Pt/C (2.77 Å, fitted from Pt/C EXAFS result), indicating that the as-prepared PtNi nanoparticles are all bimetallic alloy structures.^61−63^ Interestingly, the highest degree of Pt–Ni coordination is seen in PtNi2, while in PtNi3, the higher Ni content produces a surface that is enriched with oxidized Ni, leaving less Pt sites exposed.

**Figure 4:**
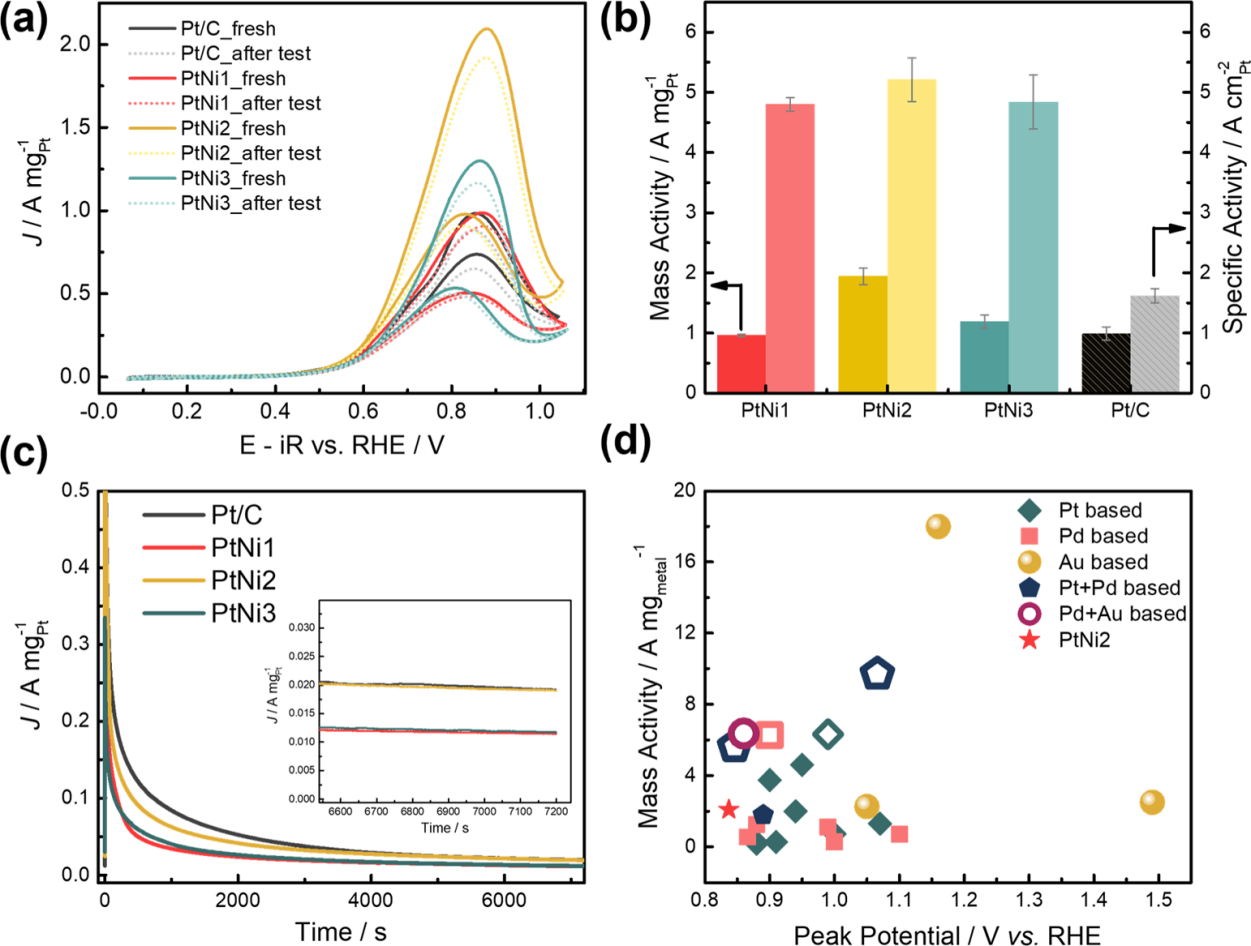
(a) CVs of Pt/C and PtNi electrocatalysts before (fresh) and after 2 h constant potential measurement at 0.9 *V*_RHE_ (after test). Electrolyte: 1 M glycerol +1 M KOH; scan rate: 50 mV s^–1^. The potentials are iR corrected after the measurements; (b) mass (left) and specific (right) activity comparison; values are averaged from three independent measurements; (c) constant potential measurements at 0.9 *V*_RHE_ (insert: zoom in of the mass current density after two h); (d) PtNi2 mass activity compared with literature values.

The electrocatalytic performances of PtNi nanoparticles toward GEOR were subsequently examined in a three-electrode cell. Figure 4a depicts the cyclic voltammetry (CV) profiles of PtNi electrocatalysts as compared to commercial Pt/C. Two separated oxidation peaks can be observed during the CV scan, with the peak in the forward scan derived from glycerol oxidation. The current density was chosen at the peak potential (0.83 *V*_RHE_) for all catalysts and normalized by the Pt mass determined from ICP–MS. Among these catalysts, PtNi2 exhibited the highest mass activity, with 1.94 A mg_Pt_^–1^ forward current density, compared to 0.96, 1.19, and 0.99 A mg_Pt_^–1^ for PtNi1, PtNi3, and Pt/C, respectively (Figure 4b). Electrochemical surface area (ECSA) values of the exposed Pt surface in all catalysts have been calculated from CO stripping, assuming that . Commercial Pt/C possesses a value of 61.2 m^2^ g_Pt_^–1^ similar to previously reported values.^64,65^ Within the three PtNi catalysts, PtNi2 showed the highest ECSA value, 37.3 m^2^ g_Pt_^–1^, compared to 20.1 and 24.6 m^2^ g_Pt_^–1^ for PtNi1 and PtNi3, respectively. Normalizing the mass activity with ECSA gives the specific activity of 4.8, 5.21. and 4.84 A cm_Pt_^–2^ for PtNi1, PtNi2. and PtNi3, respectively, suggesting a higher intrinsic activity in PtNi2.

Chronoamperometry was used to evaluate the activity and stability of the electrocatalysts as well as to study the potential-dependent GEOR product distribution. This part of the discussion will be explained in detail in the next section. The activity/stability of all of the prepared PtNi catalysts was compared at 0.9 V_RHE_ and displayed a drastic current decay at the beginning, followed by a continuous decrease over longer terms (Figure 4c). Over the 2 h test, the mass activity of PtNi2 matches that of commercial Pt/C, with 0.02 A mg_Pt_^–1^, nearly 2 times higher than that of PtNi1 and PtNi3. The CV curves after a 2 h chronoamperometry test for all catalysts are also plotted in Figure 4a. After plotting our results with the literature reported values for noble metal-based bi/trimetallic electrocatalysts (Figure 4d), it can be seen that although the magnitude of the peak current of the PtNi catalysts falls in the range of typical Pt-based materials, they require a less positive peak potential than most other similar materials.

**Figure 5:**
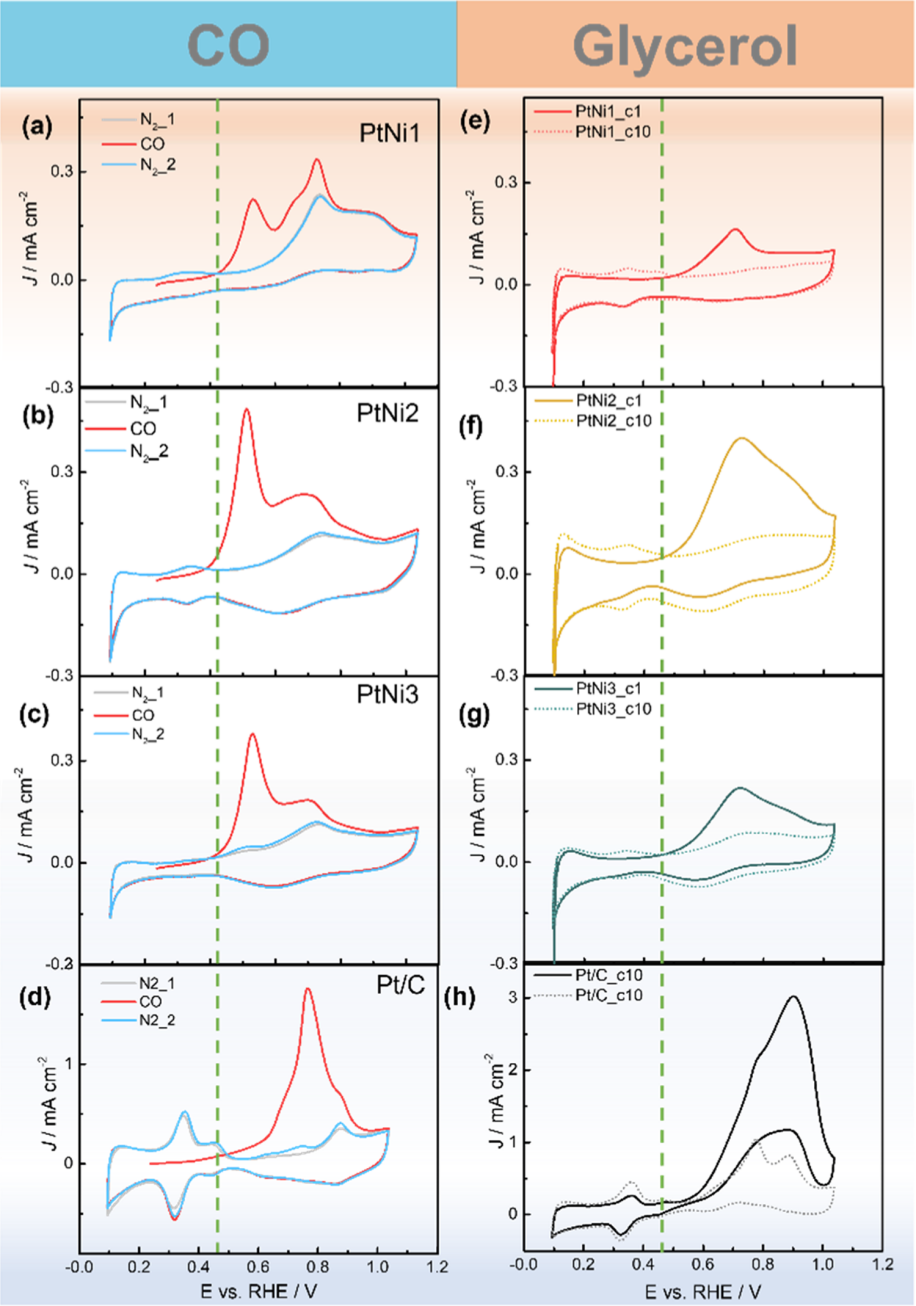
CO and glycerol stripping results of PtNi1 (a,e), PtNi2 (b,f), PtNi3 (c,g), and Pt/C (d,h), respectively. All CO stripping experiments were conducted in 1 M KOH with 50 mV s^–1^ scan rate (gray and blue curves are the current profiles recorded in N_2_ before and after the CO stripping cycle, respectively. Q_CO_ was obtained by integrating the area in between). Glycerol stripping profiles plotted here contains the 1st and 10th scan after the adsorption of glycerol molecules on the catalyst surface. Scan rate: 50 mV s^–1^. The dashed vertical lines are for aiding the comparison only.

Next, we compare CO and glycerol stripping experiments in 1 M KOH to explore the mechanism behind the different catalytic activity in the PtNi nanoparticles (Figure 5a–h). Before each stripping, the catalyst was cycled in 1 M KOH at a potential range of 0.1–1 *V*_RHE_ for 10 cycles for surface activation. This treatment will change the surface structure of the pristine PtNi nanoparticles. Since alkaline environments preclude either Pt or Ni dissolution, it is likely to cause Ni(OH)_*x*_ formation and enrichment, forming Ni(OH)_*x*_ islands on the surface.^36^ Compared to the Pt/C catalysts (Figure 5d), all three PtNi bimetallic nanoparticles exhibit two distinctive CO oxidation peaks with cathodic shifted potential, among which PtNi2 exhibit the earliest onset potential. Here, we tentatively attribute this behavior to the bifunctional mechanism and/or electronic effect mentioned earlier. Although it is difficult to separate these two effects, it is envisaged that the homogeneously distributed Pt–Ni coordination in PtNi2 has a promoting effect. Further studies to reveal the function of Ni is needed to interpret the CO stripping results.

Glycerol stripping experiments were performed to characterize possible irreversibly adsorbed intermediates during the electrochemical oxidation of glycerol (Figure S8a-d).^47^ All catalysts display distinctive oxidation-stripping peaks, which only disappear after several scans, similar to the observation by Koper et al., suggesting very strong adsorption and very slow oxidation kinetics of the corresponding adsorbate.^47^ Although the exact structure of these adsorbates remains unknown, some species have been proposed previously, which will be revisited in the next section.^48,66^Figure 5e–h shows the stripping profile comparison of the ad-species of glycerol on different PtNi catalysts. PtNi2 still exhibits the highest peak area among all PtNi catalysts, even after normalizing with ECSA (Figure S8e), indicating more glycerol adsorption sites and higher site activity, in line with the specific activity trends. Compared with the CO stripping results in Figure 5a–d, it is worth noticing that glycerol stripping profiles display a single oxidation peak, which seems to be contradictory with the bifunctional mechanism proposed for CO stripping.

**Figure 6:**
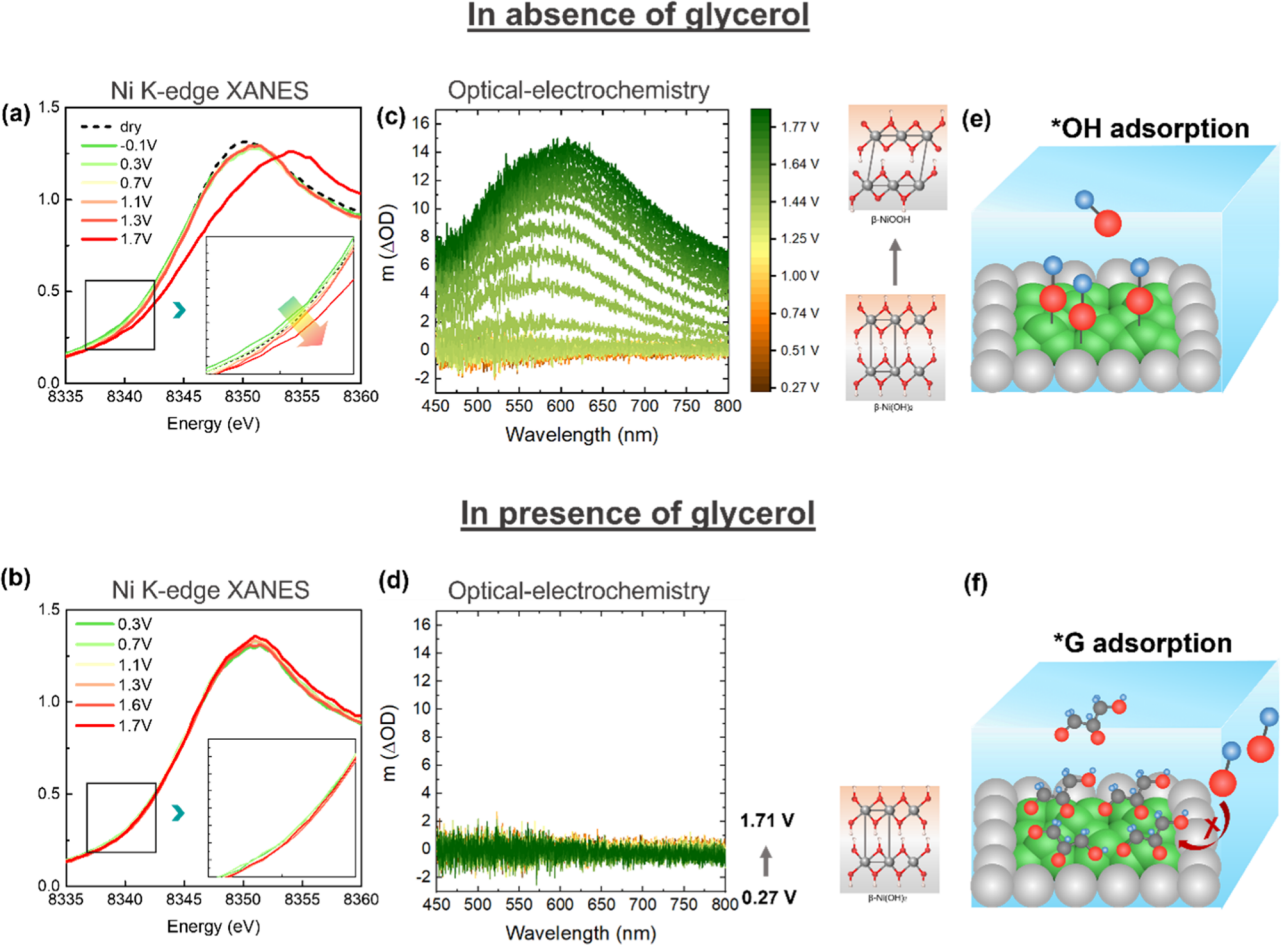
Operando XANES spectra at Ni K-edge for PtNi2 in (a) 1 M KOH and (b) 1 M KOH/0.1 M glycerol. Each potential was held for 15 min for data acquisition. The spectrum of the as-prepared sample obtained in dry N_2_ before contacting the electrolyte is shown for reference. Insets are the magnified region of the spectra between 8337 and 8443 eV. (c) Operando optical-electrochemical spectra between 450 and 800 nm during an anodic sweep in 0.1 M KOH and (d) 0.1 M KOH/0.1 M glycerol. The electrochemical data recorded during (c,d) are included in Figure S9c,d. Illustration of *OH (e) and *glycerol (f) adsorption on Ni(OH)_*x*_ islands.

To further elucidate the role of surface Ni species in the GEOR process, we then carried out systematic analysis of the nature of these species as a function of potential on the best performing PtNi2 catalyst. This was achieved using *operando* techniques characteristic to Ni chemical state changes, namely, XAS at Ni K-edge and online ultraviolet–visible (UV–vis) spectroscopy. During the *operando* XAS experiment, the potential was subsequently increased to 1.7 *V*_RHE_ in 1 M KOH or 1 M KOH/0.1 M glycerol, and XANES spectra at Pt L_3_-edge and Ni K-edge were acquired at each potential. The Ni K-edge spectra obtained during this anodic sweep are shown in Figure 6a–b, while the spectra at the Pt L_3_-edge are included in Figure S9. At the Ni–K edge, there are clear potential-dependent changes in 1 M KOH electrolyte, which are subtle in the low-potential region (<1.3 *V*_RHE_), yet became more pronounced when reaching 1.7 *V*_RHE_ (Figure 6a). This shift is due to the transition of Ni(OH)_2_ to NiOOH or even higher oxidation state NiOO, as reported previously.^67−72^ In contrast, under the same anodic sweep conditions in the presence of glycerol, the Ni XANES spectra remain nearly identical, indicating no obvious oxidation state changes (Figure 6b). The same experiment repeated at the Pt L_3_-edge does not show such distinctive behaviors in the absence and presence of glycerol (Figure S9a,b), proving that the changes are characteristic to Ni due to the strong interaction between glycerol molecules and surface Ni species, that is, Ni(OH)_*x*_ islands, as demonstrated earlier, that prevents the increase in the Ni oxidation state at more positive potentials.^36^ Next, we turned to *operando* optical absorption to determine the presence of oxidized Ni species directly in PtNi2 during electrochemical measurements in KOH and in glycerol.^72,73^ It has been shown that Ni(OH)_2_ and NiOOH display different absorption properties in the visible light range, which are directly correlated with the redox change in the catalyst.^74^ In 1 M KOH, this behavior was observed in PtNi2 during the anodic sweep from 0.27 to 1.85 V_RHE_ (Figure 6c), demonstrating the same Ni(OH)_2_ → NiOOH redox transition. However, upon adding glycerol, no optical absorption features were detected (Figure 6d), consistent with the observation in *operando* XAS. These data suggest strong interactions between glycerol and surface Ni(OH)_*x*_ islands, possibly due to the strong glycerol adsorption onto Ni(OH)_*x*_, preventing further Ni oxidation. These different surface species interactions are illustrated in Figure 6e–f. Consequently, considering the two mechanisms discussed earlier, we conjecture that Ni(OH)_*x*_ does not participate directly in the reaction via a bifunctional effect. Instead, we attribute the enhancement in PtNi catalytic activity compared to Pt/C to the modulated *CO and *OH binding on the Pt sites at the surfaces due to the strain and ligand effects induced by subsurface Ni. This modulated *CO binding on Pt surface sites makes it easier to oxidize CO, thereby resulting in the cathodic shifted potential during CO stripping measurements (Figure 5). The homogeneously distributed Pt–Ni atoms in PtNi2 induces the most optimized *CO and *OH adsorption strength, thereby displaying the highest activity among all. Next, we investigate the effect of Ni contents in determining the GEOR product selectivity.

## Product Distribution and Potential

### Reaction Mechanism and Pathways

**Figure 7:**
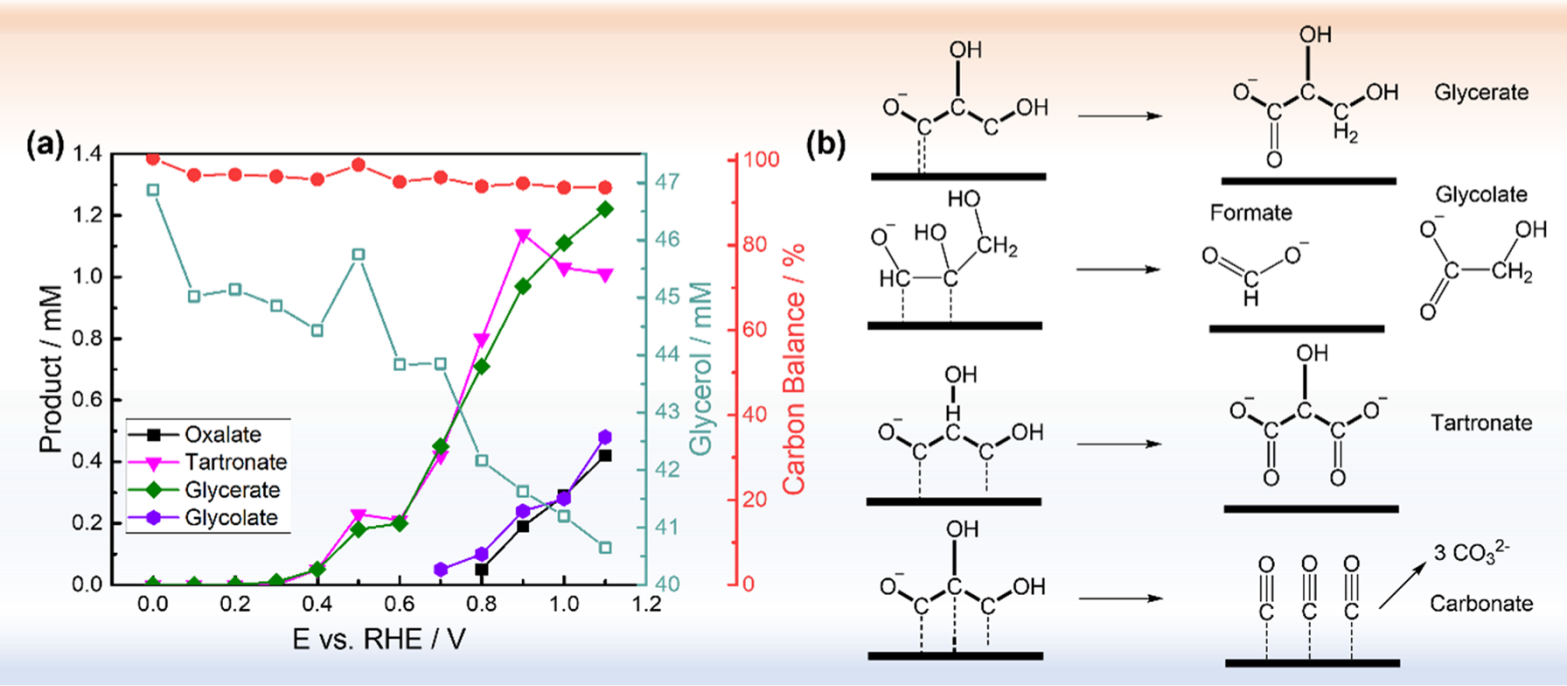
(a) Products formed at different potentials with the Pt/C catalyst detected by online sampling coupled with HPLC. Each potential was held for 10 min, and 200 μL of electrolyte containing glycerol and oxidation products were withdrawn at the end of chronoamperometry. Formate should also be produced during the reaction. However, its HPLC signal is embedded in the glycerol peak and thus cannot be quantified. (b) Proposed formation mechanism of glycerate, glycolate, formate, tartronate, and carbonate resulted from different binding geometries. The carbon balance is defined as the total carbon atoms in the solution detected by HPLC after reaction divided by that before reaction. Note that carbonate cannot be quantified with HPLC.

Prior to studying the selectivity, it is necessary to learn about the GEOR mechanism. Koper and co-workers tracked the product formation on a single crystal Pt(111) electrode in alkaline conditions using online sampling coupled with high-performance liquid chromatography (HPLC).^5^ In a more recent study, Fernandez and others have also performed similar experiments with polycrystalline Pt electrodes.^75,76^ Online sampling and HPLC experiments were also performed here on Pt/C electrocatalysts with the chromatogram profiles in Figure S10. Four main products are detected by HPLC: glycerate, tartronate, glycolate, and oxalate. The presence of formate is probed by in situ Fourier transform infrared (FTIR) spectroscopy and will be shown later in the discussion. As seen in Figure 7a, glycerate and tartronate can be formed at relatively negative potentials (0.3 *V*_RHE_) with increasing concentration at higher potentials. Contrary to the previous hypothesis, where tartronate is formed by the reoxidation of glycerate, their formation is almost simultaneous in all four testing systems, suggesting that the two reactions can take place simultaneously rather than in sequence. Glycolate, on the other hand, can only form at a potential positive than 0.7 *V*_RHE_, suggesting that the C–C bond cleavage requires higher energy than deprotonation. Although the formed carbonate cannot be quantified here by HPLC, we performed detailed in situ FTIR measurements to probe its presence and the cross-comparison between different catalysts.

Based on these prior studies, we propose the reaction mechanism on polycrystalline Pt/C in four different binding geometries, inspired by Fernandez’s work,^75^ as shown in Figure 7b, each leading to different products: (i) Binding of one primary carbon (C1) on the surface Pt atoms results in glycerate. (ii) Binding of two primary carbons (C1 and C3) results in tartronate. Since both only involve breaking C–H bonds, they can take place at relatively negative potentials. (iii) Binding of one primary carbon and one secondary carbon (C1 and C2) results in the C–C bond cleavage, which can only take place at higher potential, and produces glycolate with formate. (iv) Binding of all three carbons results in the complete glycerol oxidation toward CO_2_, which in alkaline conditions turns into carbonate (CO_3_^2–^). We anticipate that the latter scenario would involve the slowest kinetics as it would involve the formation of the surface poison, *CO.^77^ At more positive potentials, an increasing amount of oxalate, a product from the reoxidation of glycolate, has also been detected. Low quantities of lactate (below the quantification limit of the HPLC instrument) were also formed during the process (retention time ∼18 min, Figure S10), indicating the formation of low quantities of dihydroxyacetone and/or glyceraldehyde.^78^ Product distributions in the three PtNi electrocatalytic systems showed similar trends (Figure S11), which suggests that the glycerol oxidation reaction follows the same pathway as on Pt/C.

### Influence of Ni Contents

For studying the selectivity and how changing the Ni content in the PtNi bimetallic nanostructure will affect the product distribution, 1 hour chronoamperometry experiments were performed at constant potentials for Pt/C and PtNi catalysts. The current density-time traces recorded during product accumulation are shown in Figure S12. For all PtNi materials, the highest current densities are observed at 0.8 and 0.9 V_RHE_, consistent with the peak position in the CV measurements.

**Figure 8:**
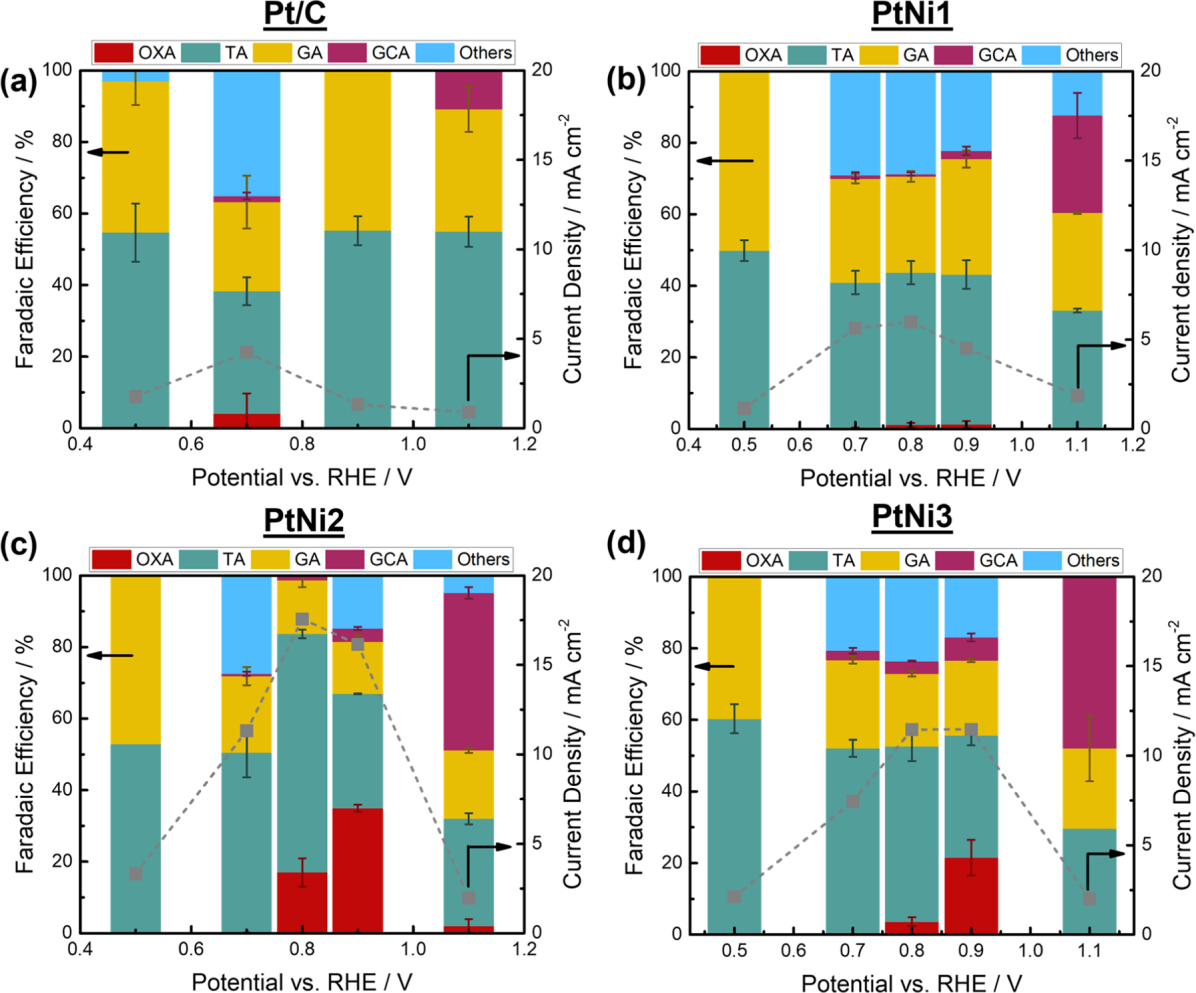
Faradaic efficiency of glycerate (GA), tartronate (TA), glycolate (GCA), oxalate (OXA), and undetectable compounds [such as carbonate, formate, etc. (others)] on (a) Pt/C, (b) PtNi1, (c) PtNi2, and (d) PtNi3 at 0.5, 0.7, 0.8, 0.9 V, and 1.1 V versus RHE. The chronoamperometry measurements were done in a custom-made three-electrode cell under static mode while the potentials were held for 1 h. The results were averaged from three independent measurements. Electrolyte: 0.1 M glycerol +1 M KOH. Electrode area: 1 cm^2^. Catalyst loading: 0.5 mg cm^–2^.

Figure 8 shows the product distribution at different potentials. At low potential (0.5 *V*_RHE_), only glycerate and tartronate can be detected. Glycolate starts to appear at 0.7 *V*_RHE,_ and its Faradaic efficiency increases with higher potential due to the higher possibility of C–C bond cleavage, in line with the online sampling coupled HPLC results in Figures 7 and S11. The Faradaic efficiency toward glycolate increases with higher Ni contents in the catalyst at all potentials above 0.7 *V*_RHE_. In particular, at 1.1 *V*_RHE_, the values changed from 19% for Pt/C to 21, 46, and 58% for PtNi1, PtNi2, and PtNi3, respectively. This trend demonstrates that higher Ni content in PtNi nanoparticles facilitates the partial C–C bond cleavage, possibly via modifying the *CO and *OH binding energy on Pt surfaces, as demonstrated previously.

**Figure 9:**
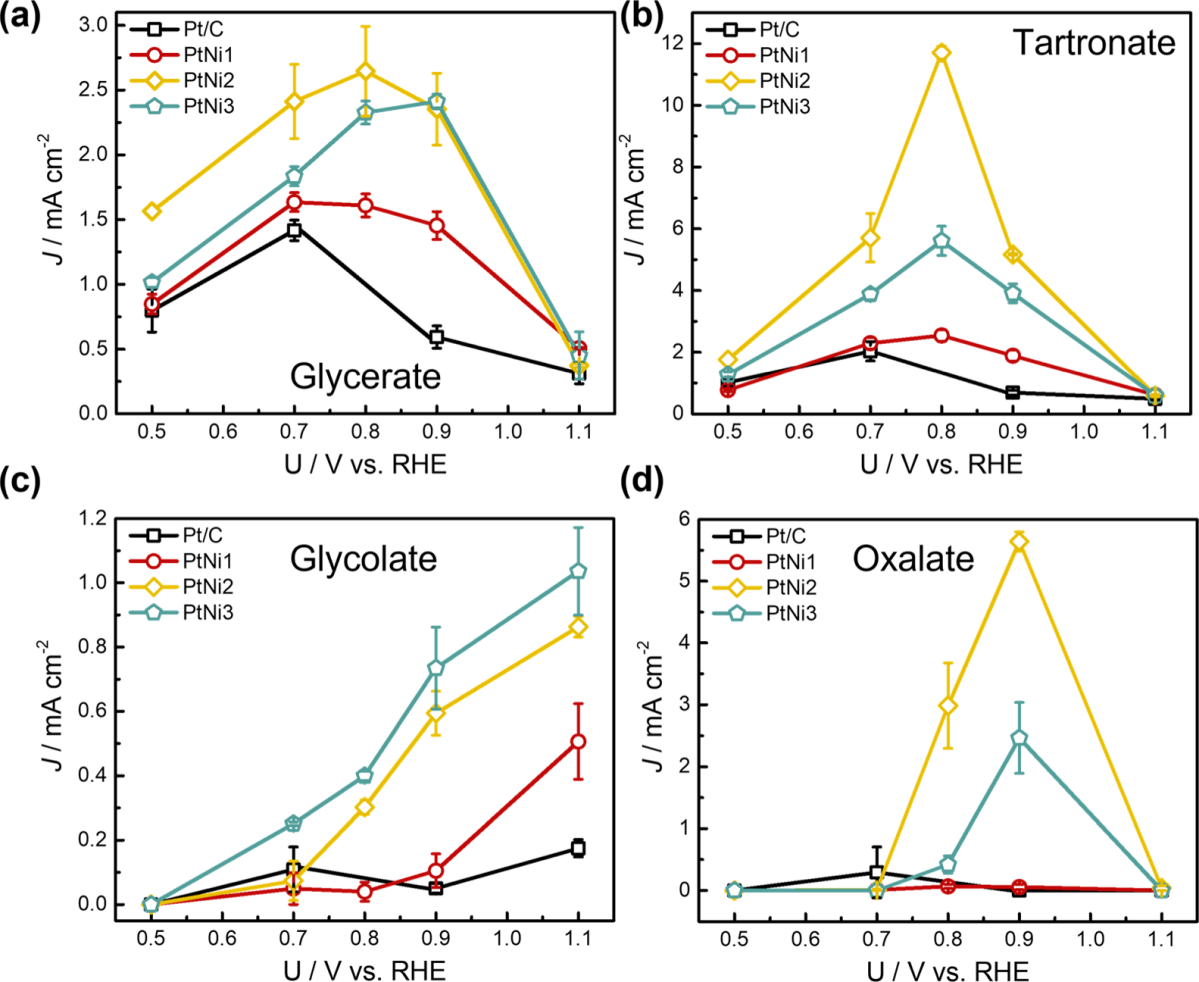
Partial current density for (a) glycerate, (b) tartronate, (c) glycolate, and (d) oxalate on Pt/C and PtNi electrocatalysts derived from the Faradaic efficiency in Figure 8, based on the equation *J*_p_ = *J*_total_ × FE %. The current density at the end of the 1 h period for each potential was used as the total current density.

Figure 9a displays the partial current density for glycerate, tartronate, glycolate, and oxalate for all four catalysts. For glycerate production, all four catalysts display similar potential dependent behavior, with the highest selectivity obtained in the middle of the potential range studied (0.7–0.9 *V*_RHE_). This result implies that the presence of Ni is unlikely to alter the reaction pathway toward glycerate. For tartronate, the highest selectivity is seen at 0.8 *V*_RHE_, with PtNi2 showing the highest partial current density (11.7 mA cm^–2^) and Faradaic efficiency (67%) (Figure 9b). Similar to the observation for Faradaic efficiency, the partial current density toward glycolate increases with higher potential. Increasing the Ni contents in PtNi2 and PtNi3 results in the formation of glycolate at much more negative potential than PtNi1 and Pt/C (Figure 9c), further demonstrating the effect of higher Ni contents in promoting the partial C–C scission.

**Figure 10:**
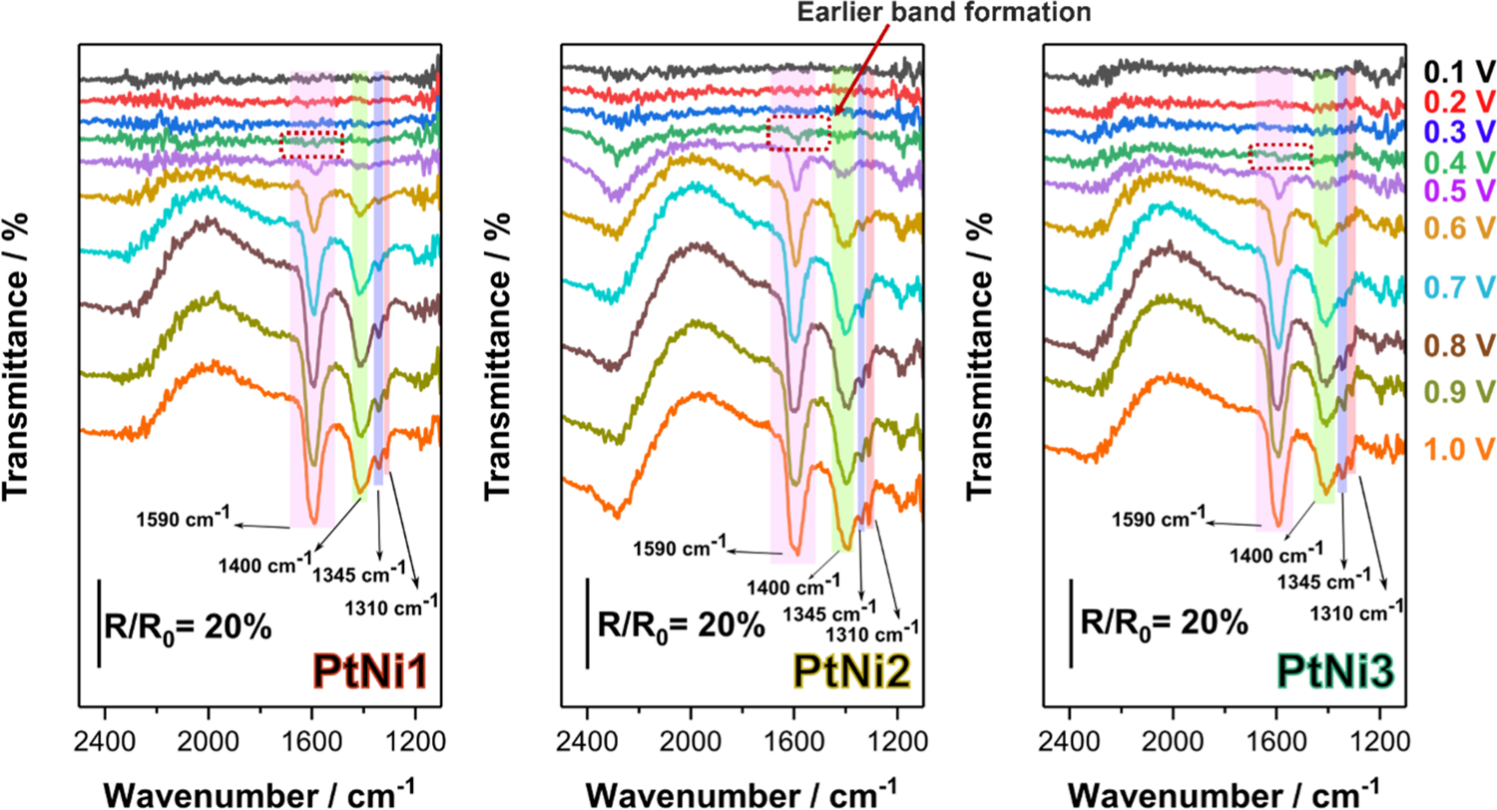
In situ FTIR for different compositions of PtNi nanoparticles (left: PtNi1; mid: PtNi2; right: PtNi3) obtained in 0.1 M glycerol +1 M KOH. Spectra are composed of 256 interferograms with 4 cm^–1^ resolution. Reference spectra acquired at 0.1 *V*_RHE_.

In the following, we rationalize the observations in bulk oxidation tests using in situ FTIR with mechanistic analysis at the catalyst surface. The experimental configurations can be found in the Supporting Information and a previous report.^79^Figure 10 shows the in situ FTIR results for the three different PtNi compositions. The results show the same set of bands, namely, (i) a band at ∼ 1590 cm^–1^, which is associated with several carbonyl-containing compounds (glycerate, oxalate, and formate, etc.) and (ii) bands at ∼ 1400, ∼1345, and 1310 cm^–1^ related to carbonate (CO_3_^2–^), formate, and oxalate, respectively.^75,76,80,81^ While a weak band at 1590 cm^–1^ appears at 0.4 V_RHE_ for PtNi2, this band only appears at 0.5 V_RHE_ for PtNi1 and PtNi3, as well as Pt/C reported in our previous study,^80^ indicating PtNi2 can catalyze the reaction at a more negative potential than PtNi1, PtNi3, and Pt/C, in line with the negatively shifted onset potential observed in CO stripping experiments. Looking at the relative intensities of the carbonyl (∼1590 cm^–1^) and carbonate (∼1400 cm^–1^) bands, PtNi2 has a higher relative intensity for the carbonate band compared to PtNi1 and PtNi3. Combined with the hypothesis presented in Figure 7b, it can be deduced that the PtNi2 surface can effectively catalyze the complete C–C bond cleavage in the three–carbon binding geometries (scenario iv), resulting in lower CO poisoning and higher stability. In addition, the oxalate band at higher potential is also more intense than the formate band in PtNi2 compared to those in PtNi1 and PtNi3, indicating that more glycolate has been converted than being produced by the glycerol C–C bond cleavage. These results are in perfect agreement with the HPLC and partial current density results in Figures 8 and 9d.

From the above results, it can be concluded that the PtNi nanoparticles’ Ni content plays a significant role in tuning the reaction pathways and kinetics. Adding more Ni to the electrocatalyst will reduce the barrier to the partial C–C cleavage and hence increase selectivity toward glycolate and formate. Among all three catalysts, PtNi2 shows faster GEOR kinetics, which drives the reaction at a potential as low as 0.4 V_RHE_. In addition, it can also catalyze the most sluggish complete C–C scission to carbonates. It is hypothesized that the uniformly coordinated Pt–Ni structure in PtNi2 induces the most optimized *CO and *OH adsorption strength, thereby displaying the highest activity and C–C bond cleavage ability. As mentioned earlier, Pt-based materials can catalyze the breaking of C–C bond (e.g., pure Pt materials) but generate CO and other intermediates, poisoning the surface.^75,76^ However, in the case of PtNi2, the higher C–C bond cleavage ability is associated with greater poisoning tolerance, which significantly improves the reaction activity and stability.

## Conclusions

**Scheme 1:**
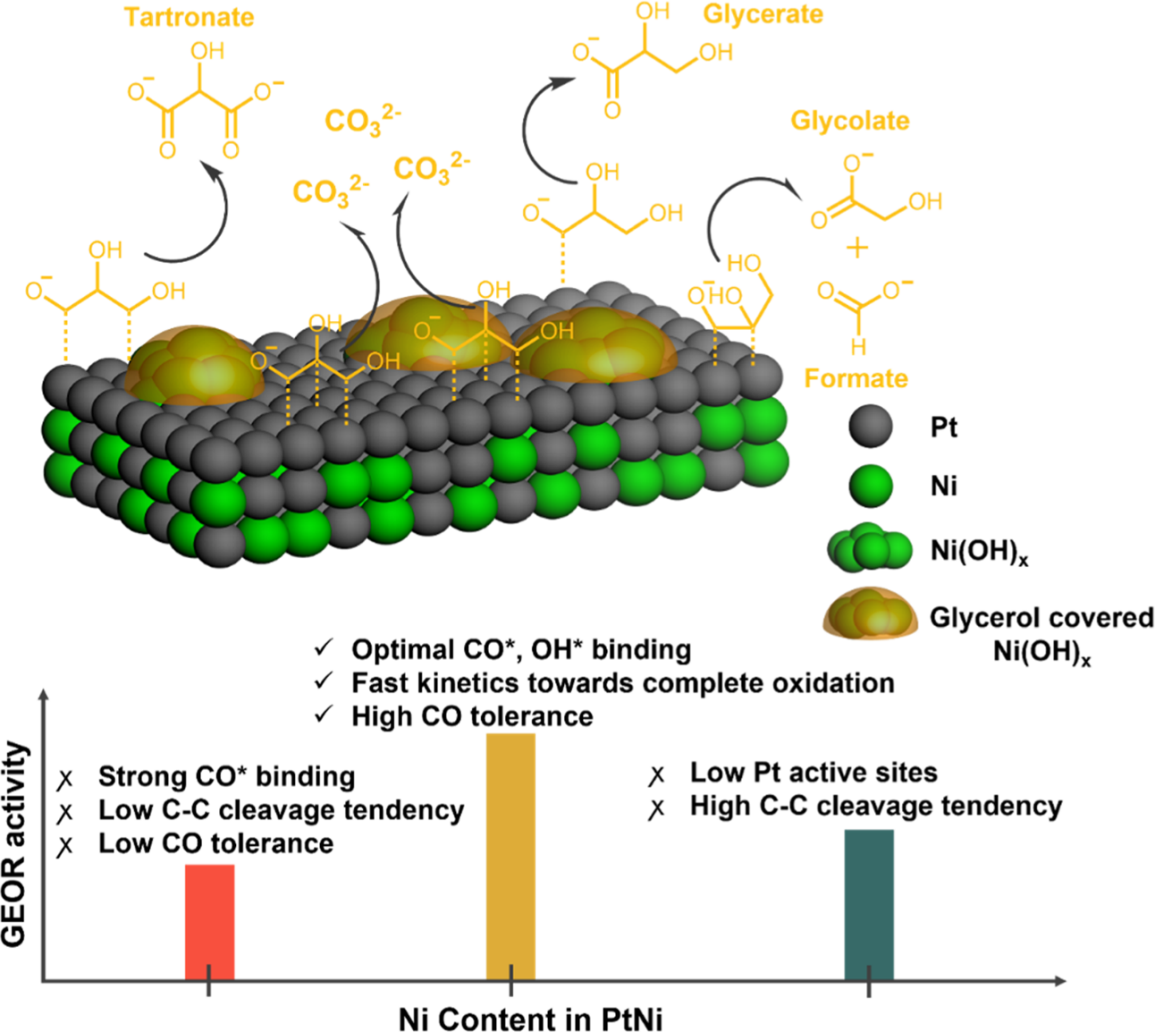
Illustration of the Electronic Effect in PtNi Electrocatalysts (Green: Ni, gray: Pt) Under Potential less than 1 *V*_RHE_ The surface Ni(OH)_x_ islands at this condition are covered entirely by glycerol adsorbates, therefore do not contribute to the GEOR process.

In summary, three PtNi bimetallic catalysts were synthesized, with increasing Ni contents from PtNi1 to PtNi3. Among them, PtNi2 exhibits the most homogeneous coordinated Pt–Ni structure and the highest glycerol oxidation activity. Previous studies have hypothesized that this enhanced activity is either (i) a result of the bifunctional mechanism at adjacent Pt and Ni sites^30−37^ or (ii) due to the modified *CO and *OH binding energy on Pt surfaces.^38−41^ Here, we performed stripping and *operando* spectroscopic techniques to shed light on the role of surface Ni species. Under alkaline conditions, the surface Ni atoms are likely to form Ni(OH)_*x*_ islands; our evidence suggests that these islands are poisoned by glycerol molecules, thus blocking the sites for *OH adsorption. Based on this observation, we conjecture that the bifunctional effect is inoperative. Instead, the presence of Ni will modulate the electronic structure of surface Pt atoms, thus weakening the *CO and *OH binding energy. The Pt–Ni structure in PtNi2 induces the most optimized *CO and *OH adsorption strength on Pt sites, thereby displaying the highest activity among all catalysts in this study. As for selectivity, increasing Ni content in PtNi nanoparticles does not alter the selectivity toward glycerate and tartronate to a large extent but significantly promotes the partial C–C bond cleavage toward glycolate formation. In particular, the best performing PtNi2 is able to completely break C–C bonds while maintaining high resistance to surface poisoning, representing a crucial aspect in Pt-based electrocatalyst design. These findings have been summarized in Scheme 1. The result from this work not only provides insights into the correlation between atomic-scale structure and electrocatalytic activity of bimetallic catalysts but also, for the first time, clarifies the role of Ni content on the GEOR kinetics and reaction pathway, allowing for the rational development of bimetallic catalysts toward highly efficient, selective, and stable glycerol oxidation reaction.

Finally, on the basis of our spectroscopy analysis on Ni redox species, we conjecture that the reason that pure Ni(OH)_*x*_ requires such high overpotentials for glycerol oxidation, relative to platinum group metals, is because it is poisoned by the glycerol itself; at these high overpotentials, highly reactive *OH species are formed which overoxidize the glycerol, cleaving C–C bonds and forming products such as formate which has a lower economic value than C_2+_ products. Our future studies will focus on improving the stability of the catalysts by investigating the catalysts’ morphological and structural evolution during the reaction, as well as how to oxidize glycerol to valuable C_2+_ products without resorting to expensive and scarce platinum group metals.

## Supporting Information Available

Experimental section on catalyst synthesis, material characterizations, electrochemical experiments, operando XAS, optical-electrochemistry, product analysis, in situ FTIR, XRD, low-magnification HAADF-STEM, XPS, and ICP–MS values, XAS fitting results, glycerol stripping and online product analysis, and chronoamperometry profiles (PDF)
