# Supplementary material for: Role of Ni in PtNi Bimetallic Electrocatalysts for Hydrogen and Value-Added Chemicals Coproduction via Glycerol Electrooxidation
Source: ACS Catal. 2022 Nov 10;12(23):14492–506. doi: 10.1021/acscatal.2c03907 (PMC9724082; doi:10.1021/acscatal.2c03907)
Supplement: Supplementary file 1 — cs2c03907_si_001.pdf [file cs2c03907_si_001.pdf]

The role of Ni in PtNi bimetallic electrocatalysts for hydrogen and value-added chemicals co-production *via* glycerol electrooxidation

Hui Luo,[a] Victor Y. Yukuhiro,[b] Pablo S. Fernández,[b] Jingyu Feng,[a,c] Paul Thomphson,[d] Reshma R. Rao,[e] Rongsheng Cai,[f] Silvia Favero,[a] Sarah J. Haigh,[f] James R. Durrant,[g,h] Ifan E. L. Stephens,[e] Maria-Magdalena Titirici[a,i]**

a. Department of Chemical Engineering, Imperial College London, South Kensington Campus, SW7 2AZ, London, UK

b. Chemistry Institute and Center for Innovation on New Energies, State University of Campinas, P.O. Box 6154, 13083-970 Campinas, São Paulo, Brazil

c. School of Engineering and Materials Science, Queen Mary University of London, E1 4NS, London, UK

d. XMaS CRG, ESRF, 71 avenue des Martyrs, 38000 Grenoble, France

e. Department of Materials, Imperial College London, South Kensington Campus, SW7 2AZ, London, UK

f. School of Materials, University of Manchester, Oxford Road, Manchester M13 9PL, UK

g. Centre for Processable Electronics, Imperial College London, SW7 2AZ, London, UK

h. Department of Chemistry, Imperial College London, South Kensington Campus, SW7 2AZ, London, UK

i. Advanced Institute for Materials Research (WPI-AIMR), Tohoku University, 2-1-1 Katahira, Aobaku, Sendai, Miyagi, 980-8577, Japan.

Corresponding Author

* i.stephens@imperial.ac.uk (Ifan E. L. Stephens)

*m.titirici@imperial.ac.uk (Maria-Magdalena Titirici)

Experimental methods

Synthesis of PtNi bimetallic electrocatalysts

The three type of PtNi bimetallic nanoparticles were synthesized by a one-step solvothermal method in dimethylfomamide (DMF) adapted from Strasser *et al.*[1] In brief, precursor solutions were obtained by completely dissolving 4 mM $Pt(acac)_2$/3 mM $Ni(acac)_2$, 4 mM $Pt(acac)_2$/10 mM $Ni(acac)_2$, and 4 mM $Pt(acac)_2$/28 mM $Ni(acac)_2$ into 25 mL DMF, respectively. Then, the solutions were transferred into a glass tube within a Teflon-lined stainless-steel autoclave and heated at 120 °C for 42 hours. After the long time reaction, PtNi nanoparticles were synthesized. Before washing the particles, 20 mg Vulcan XC-72 carbon was added to the mixing solution and ultrasonicated for 30 min to serve as a support for the as produced nanoparticles. The PtNi bimetallic catalysts supported on carbon were then copiously washed with ethanol/18.2 MΩ di-ionized water two times, followed by washing with acetic acid at 60 °C for 12 hours under stirring for surface cleaning. The cleaned catalysts were then washed again 3 times with ethanol/water, freeze dried, and annealed at 200 °C for 2 hours under N_2 flow. The collected final products were denoted as PtNi1, PtNi2 and PtNi3, respectively.

Materials Characterization

High-resolution transmission electron microscopy (HR-TEM) images were obtained by a FEI Titan 80/300 at an operating voltage of 300 kV. High-angle annular dark field scanning transmission electron microscopy (HAADF-STEM) images, energy-dispersive X-ray (EDX) mapping and electron energy loss spectroscopy (EELS) were carried out with a probe aberration-corrected analytical electron microscope (JEOL ARM200F) in STEM mode with electron beam voltage set at 200 kV. The X-ray diffraction (XRD) patterns were performed using PANanalytical's X'PERTPRO X-ray diffractometer with Ni filtered Cu Kα radiation and an X'Celerator multistrip detector. The scanned two theta ranges from 15 to 90 at 3 /min. To reduce the noise in the final signal, the sample holder was rotating at 90 per second. The X-ray photoelectron spectroscopy (XPS) of all catalysts were conducted on a Thermo Scientific Nexsa XPS system with a monochromatic Al Ka X-ray source. C 1s spectra were used to calibrate the peaks in all samples. Pt and Ni contents in each catalyst were quantified with inductively coupled plasma mass spectrometry (ICP-MS) using Agilent Nu Instruments with Nu Plasma multiple collectors. Before analyzing, the materials of ~ 5 mg were carefully weighted and digested by 8 mL aqua regia using a MARS 6 microwave at 1200W for 15 min. the solutions were filtered and diluted with 2% HNO3 and 1% HCl matrix to low metal contents (< 500 ppb). For each sample, two duplicates were measured for accuracy with the error below 5%. The reported values for all samples are the average of the two duplicates. for Ex-situ Pt L_3 edge and Ni K edge X-ray adsorption spectroscopy (XAS) measurements were performed at XMaS (BM28) beamline in European Synchrotron Radiation Facility (ESRF) and I20-EDE beamline in diamond light source Pt and Ni foils with 25 μm thickness were used to calibrate the Si (111) double-crystal monochromator and used for data collection as a Pt standard afterwards. Additional reference compounds such as platinum (IV) oxide (PtO_2), nickel oxide (NiO) and nickel hydroxide ($Ni(OH)_2$) were also measured. The XAS data was processed with Athena and Artemis software, and the S_0^2 for both Pt and Ni edge samples was obtained by fitting the Pt and Ni foils.

Electrochemical Experiments

Electrochemical experiments with product analysis were carried out in 3-electrode setup in one compartment PTFE cell. 5mm diameter rotating disk electrode (RDE) was used for activity measurements, and catalyst coated carbon paper (Freudenberg H23) was used for product analysis. The catalyst inks were prepared by dispersing 4mg of catalysts in 1400 μL water, 528 μL ethanol and 71 μL 5 wt % Nafion solution, followed by probe sonication for 10 min. For

RDE measurement, 10 μL ink was coated onto the glassy carbon rod on RDE. For carbon paper electrode (1 cm^2), the inks were spray coated onto the carbon paper to achieve 0.5 mg cm^{-2} catalyst loading.

The electrolyte was prepared by dissolving KOH (suprapur, Merck) in ultrapure water (18.2 MΩ), and diluting pure glycerol (99.5%). A Pt mesh counter and Hg/HgO reference electrode were employed. The reference electrode was calibrated regularly against the RHE scale by measuring the hydrogen evolution/oxidation potential at a Pt rotation disk electrode (RDE) in H_2 saturated electrolyte. N_2 gas (zero grade N4.8, BOC) was bubbled for 30 min before each measurement.

The activity and stripping experiments were carried out using RDE (5 mm diameter glassy carbon, 0.196 cm^2) and an Autolab potentiostat. The electrolyte is 1 M glycerol + 1 M KOH. Cyclic voltammetry for all catalysts was performed between 0.1 – 1.1 V vs. RHE at scan rate of 50 mV s^{-1}. Chronoamperometry was performed at 0.9 V vs. RHE for 2 hours to investigate the short-term stability. Mass and specific activities were compared at the peak potentials for all catalysts.

The product analysis experiments were conducted using the 1 cm^2 catalyst coated carbon paper as the working electrode, along with a Pt mesh counter and Hg/HgO reference electrode in a 20 mL volume one compartment PTFE cell. For each measurement, 10 mL of 0.1 M glycerol + 1 M KOH electrolyte was used, and the product solution was collected after 1 hour electrolysis at constant potential of 0.5, 0.7, 0.8, 0.9 and 1.1 V vs. RHE, respectively.

CO stripping was used to determine the electrochemical surface area (ECSA) and surface catalytic characteristics. The electrode was polarized at 0.2 V *vs* RHE and CO(g) was bubbled in the cell for 15 minutes, then N_2(g) was bubbled for 30 minutes, followed by a cyclic voltammetry in a range of 0.1 V – 1.1 V at 50 mV.s^{-1}. ECSA values were evaluated from the CO oxidation charge of the linear background corrected first stripping cycle under nitrogen atmosphere. This measured charge Q_{CO} was then normalized using the theoretical value of Q_{CO}^{theo}=420 μC cm^{-2} for a two-electron transfer assuming the oxidation of one CO to CO_2 per Pt atom.[2]

Glycerol stripping measurements were performed by bringing the working electrode into contact with 0.1 M glycerol + 1 M KOH electrolyte and keeping the potential at 0.2 V vs. RHE for 5 mins, followed by flushing out the electrolyte with excessive amount of 1 M KOH for 30 min. 8 CVs are then performed to ensure all the glycerol adsorbed on the catalyst's surface has been oxidized due to the slow kinetics.[3]

Operando XAS

The operando XAS measurements were conducted at the i20-EDE beamline in diamond light source, using a self-designed electrochemical cell made from PEEK. Before measuring the materials under electrochemical condition, XAS spectra were recorded under dry N_2, and later used as the reference. The potential was controlled with a Ivium OctoStat200 potentiostat and applied between a platinum wire (*i.e.* the counter electrode) and the sample (*i.e.* the working electrode). The potential at the working electrode was measured with respect to an Ag/AgCl reference electrode saturated with KCl, which was calibrated against RHE prior coming to the beamline. During the measurement, N_2-purged electrolytes were circulated through the cell to prevent air contamination. The electrode potential was incrementally increase. All the potentials were iR corrected. Each potential was held for 15 mins during the X-ray data acquisition.

Optical-electrochemistry

For optical-electrochemistry measurements, samples were deposited on ~1cm x 1cm area of FTO substrates. Measurements were made in a three-electrode cell using a home-built optical spectroscopy setup. A stabilized 10mW tungsten-halogen light source from Thorlabs (SLS201L) was used with a collimating add on (SLS201C). The light emitted from the lamp was transmitted through the sample and collected using a 1 cm diameter liquid light guide (Edmund optics). Light transmitted to the spectrograph was first collimated and refocused using two 5 cm planoconvex lenses (Edmund) in order to optimally match the optical components of the spectroscope (Kymera 193i, Andor), CCD camera (iDus Du420A-BEX2-DD, Andor). The detector was maintained at -80 ^{0}C during the measurements to ensure high signal-to-noise ratio. An Ivium Vertex potentiostat was used. Data acquisition was facilitated by a custom-built LabView software. Measurements were made in potentiostatic mode. The equilibration time at each potential was 10 seconds. This was followed by measurement of the optical spectra. At each potential, 30 averages of the spectra were taken (each spectral acquisition takes ~30 ms), before moving to the next potential. Simultaneously, the current was measured at each potential using the Ivium Vertex potentiostat. The potential was measured with respected to a Ag/AgCl reference electrode (saturated KCl), which was calibrated versus the reversible hydrogen electrode. A Pt mesh was used as the counter electrode.

Product Analysis

High-performance liquid chromatography (HPLC) was performed with an Agilent 1260 Infinity II LC System to detect the liquid products produced during the electrochemical oxidation of glycerol. Different compounds were separated with an Aminex HPX-87H column kept at 65 °C and detected by a refractory index detector at 50 °C. The mobile phase was 5 mM H_2SO_4 with a flow rate of 0.4 mL min^{-1}, and the injection volume was 1 µL. 160 µL reaction products were collected after 10 min chronoamperometry continuously for the online sampling experiments. For faradaic efficiency and partial current density determination, 200 µL reaction products were analyzed after 1 hour chronoamperometry at 0.5, 0.7, 0.8, 0.9 and 1.1 V_{RHE}, respectively.

***In situ* FTIR**

In situ Fourier Transform Infrared (FTIR) experiments were performed using a Shimadzu IR-Prestige-21 spectrometer with a mercury cadmium telluride (MCT) detector. A spectroelectrochemical cell equipped with a CaF_2 prism placed on top of a specular reflection accessory (Veemax II, Pike Technologies) was used for the measurements. More details can be found elsewhere.[4]

The preparation of the working electrode (WE) consists of depositing 60 µL of the nanoparticle ink on the surface of the vitreous carbon electrode. Prior to each experiment, in a three electrodes electrochemical cell containing 1 M KOH, a CO stripping were performed.

After the preparation of the electrode, it was polarized at 0.1 V, and then inserted in the spectroelectrochemical cell containing 0.1 M glycerol + 1 M KOH, put in contact with the electrolyte and pressed onto the prism to form the thin layer. The spectra were obtained during a stepped chronoamperometry in a range of 0.1 V – 1 V. At each potential, 256 interferograms with a resolution of 4 cm^{-1} were acquired. The spectra were plotted as the ratio R/R_0, where R_0 is the reference spectra obtained at 0.1 V and R is the spectra in function of potential. All measurements were performed in D_2O and all potentials were applied *vs* RHE.

The carbon balance, taken glycerol as an example, is expressed as:[5]

$$Carbon\ balance = \frac{3M_{C3} + 2M_{C2} + M_{C1} + 3M_{Gf}}{3M_{Gi}} \times 100\%$$

Where M_{Gi} and M_{Gf} are the initial and final moles of glycerol, M_{C1}, M_{C2} and M_{C3} are the moles of C_1, C_2 and C_3 products, respectively.

While Faradaic efficiency (FE%) is determined based on:[6]

$$Faradaic\ efficiency\ (\%) = \frac{mole\ of\ product\ formed\ \times nF}{total\ charge\ passed} \times 100\%$$

Where F is the Faradaic constant (96,485 C mol^{-1}) and n is the electron transfer number.

Supporting Figures

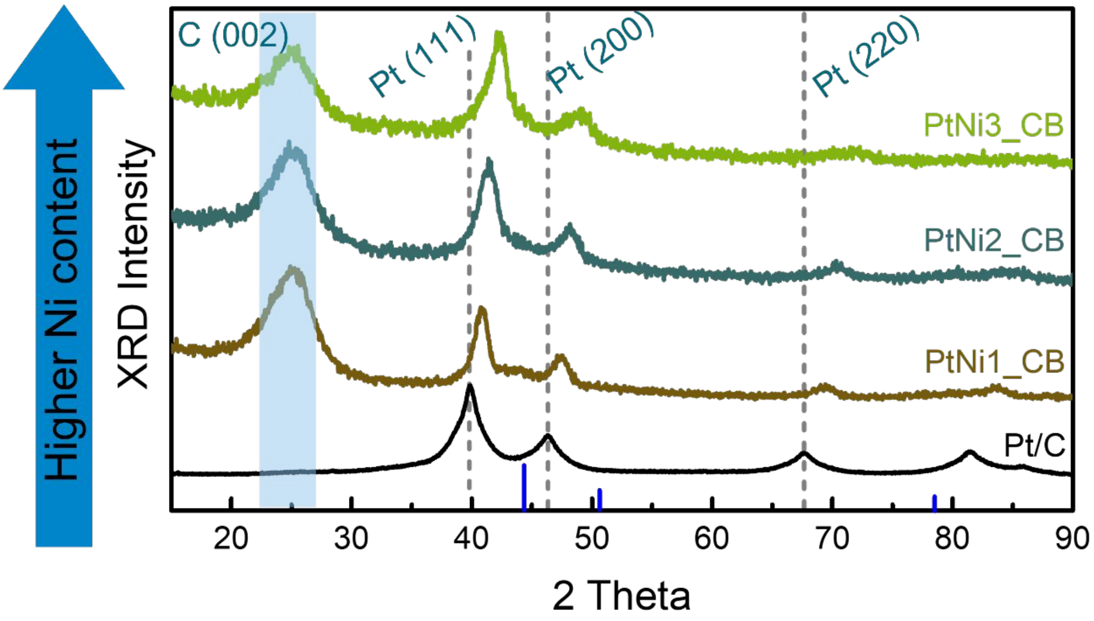

Figure S1 *XRD profiles of Pt/C and PtNi nanoparticles on carbon black (CB). Reference profile for Ni is shown by the blue lines (PCPDFWIN #040-850).*

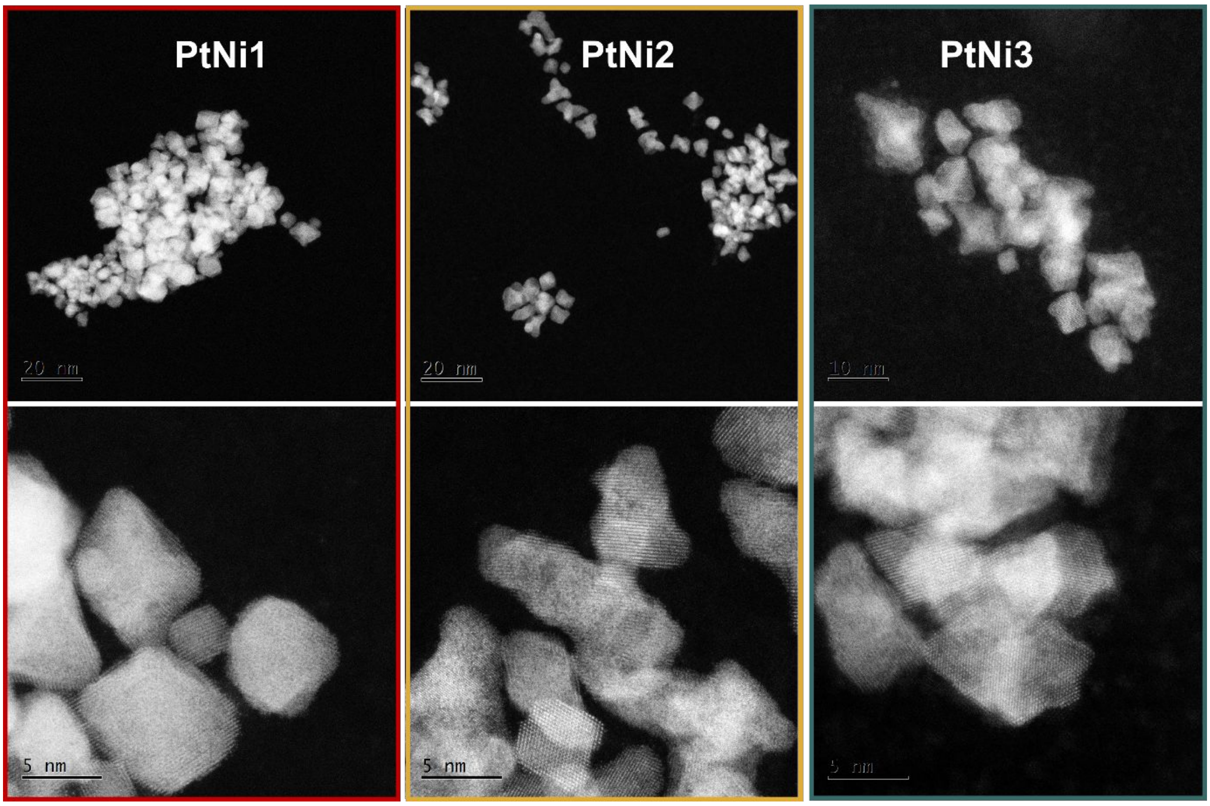

Figure S2 *HAADF-STEM images of PtNi1, PtNi2 and PtNi3.*

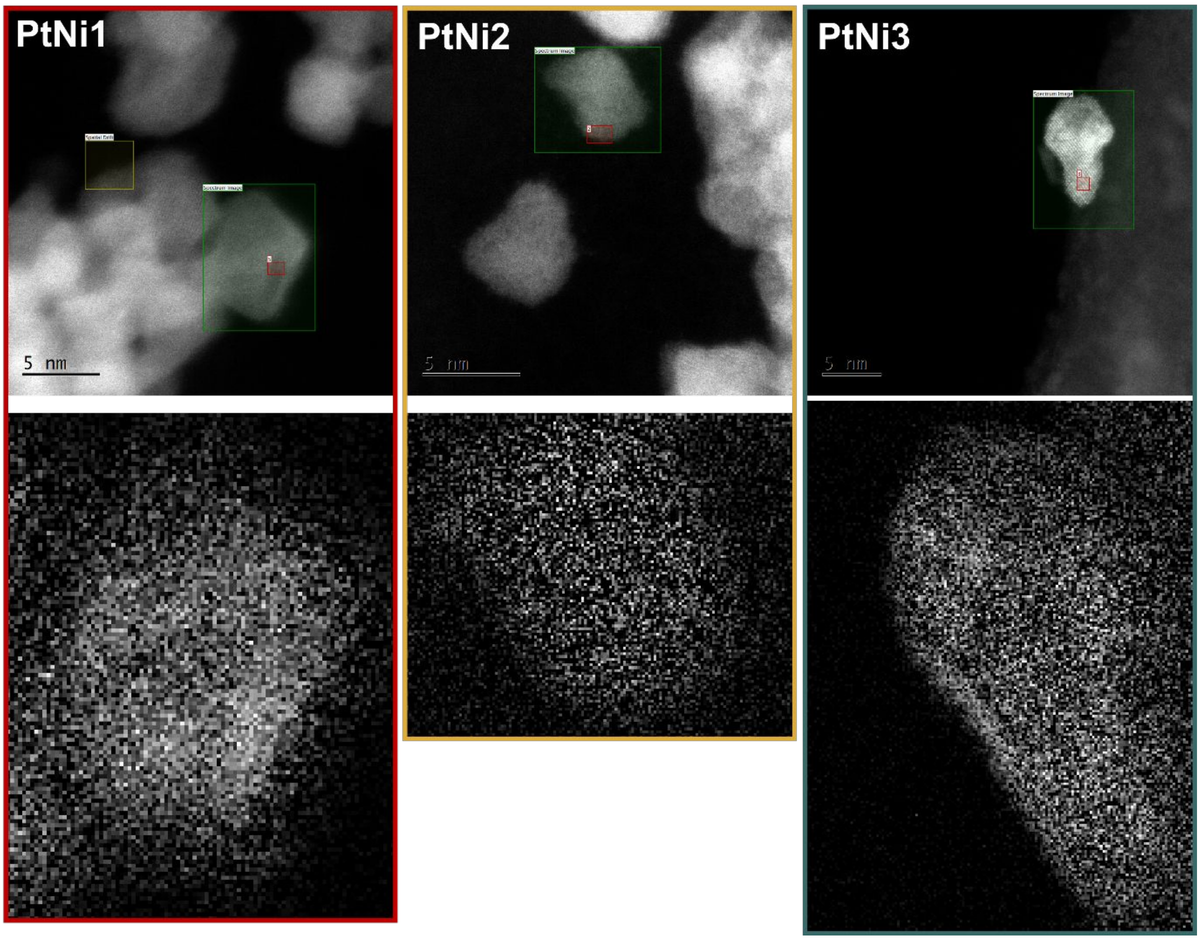

Figure S3 *HAADF-STEM survey images for PtNi, PtNi2, and PtNi1-3 with the corresponding Ni $L_{2,3}$-edge EELS maps below (~855 eV energy loss) for the regions indicated by the green rectangle. The yellow square in PtNi1 is for drift correction during spectral image acquisition. Red rectangles were used for imaging the spectral data.*

Table S1 *Pt and Ni metal composition in three PtNi nanoparticle samples derived from XPS and ICP-MS*

	**XPS**			**ICP-MS**		
Sample	Pt at/wt %	Ni at/wt %	Pt/Ni surface	Pt wt %	Ni wt %	Pt/Ni bulk (wt)
PtNi1	1.5/18.6	0.7/2.6	2.2 (at) 7.3 (wt)	27.1	3.3	8.2
PtNi2	1.1/14.8	0.7/2.7	1.7 (at) 5.6(wt)	31.4	7.3	4.3
PtNi3	1/13.2	0.9/3.4	1.2 (at) 3.9 (wt)	29.9	18.6	1.6

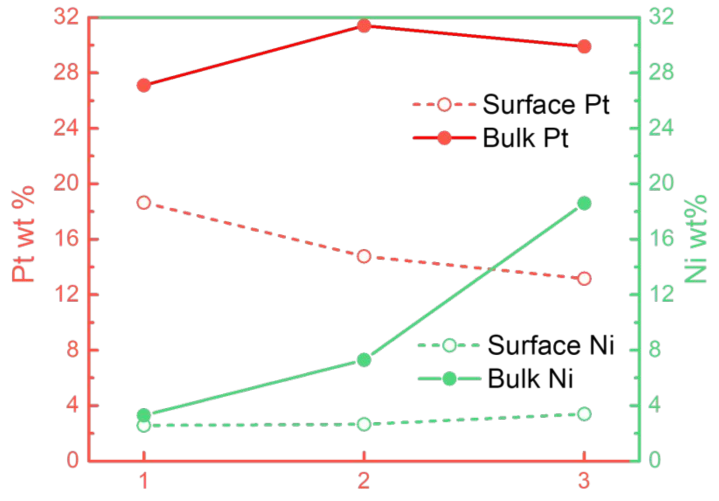

Figure S4 *Pt and Ni content difference between surface (measured by XPS) and bulk (measured by ICP-MS). Raw data in Table S1.*

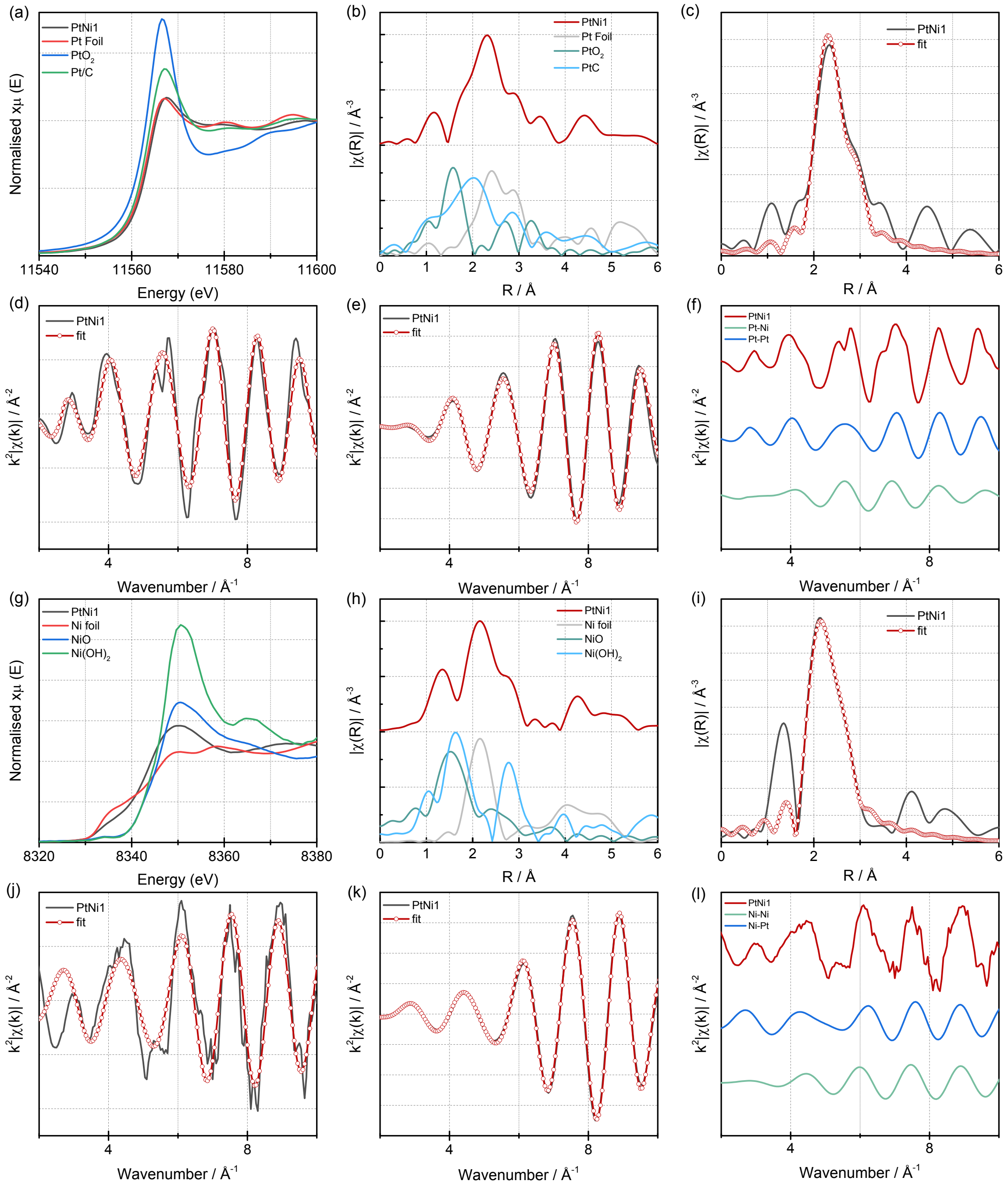

Figure S5 PtNi1 Pt L_3-edge: *a) XANES and b) EXAFS r-space, EXAFS fitting results in c) r-space, d) k-space, e) q-space and f) k-space contribution of each path.* ***PtNi1 Ni K-edge:*** *g) XANES and h) EXAFS r-space, EXAFS fitting results in i) r-space, j) k-space, k) q-space and l) k-space contribution of each path.*

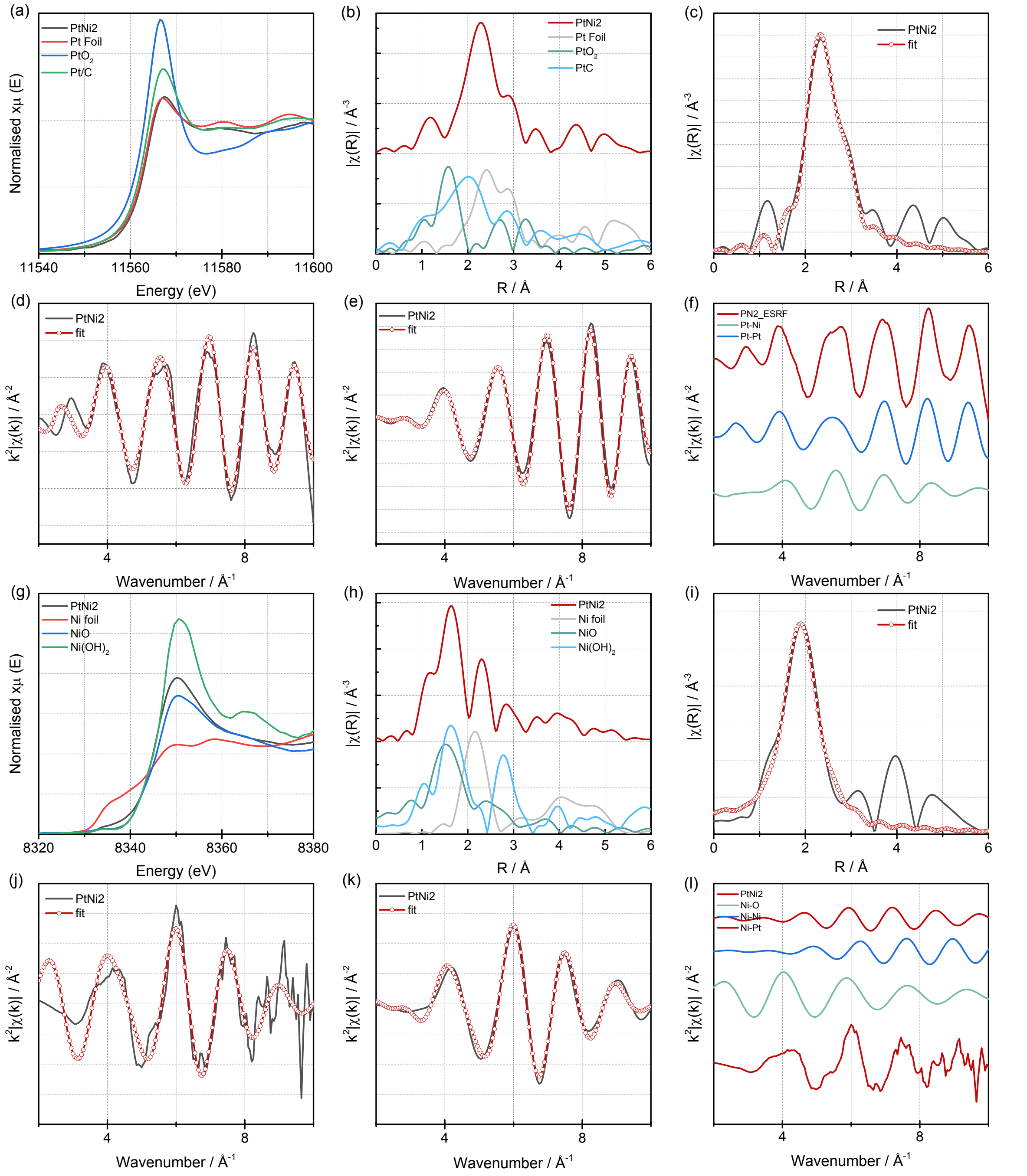

Figure S6 PtNi2 Pt L_3-edge: *a) XANES and b) EXAFS r-space, EXAFS fitting results in c) r-space, d) k-space, e) q-space and f) k-space contribution of each path.* ***PtNi2 Ni K-edge:*** *g) XANES and h) EXAFS r-space, EXAFS fitting results in i) r-space, j) k-space, k) q-space and l) k-space contribution of each path.*

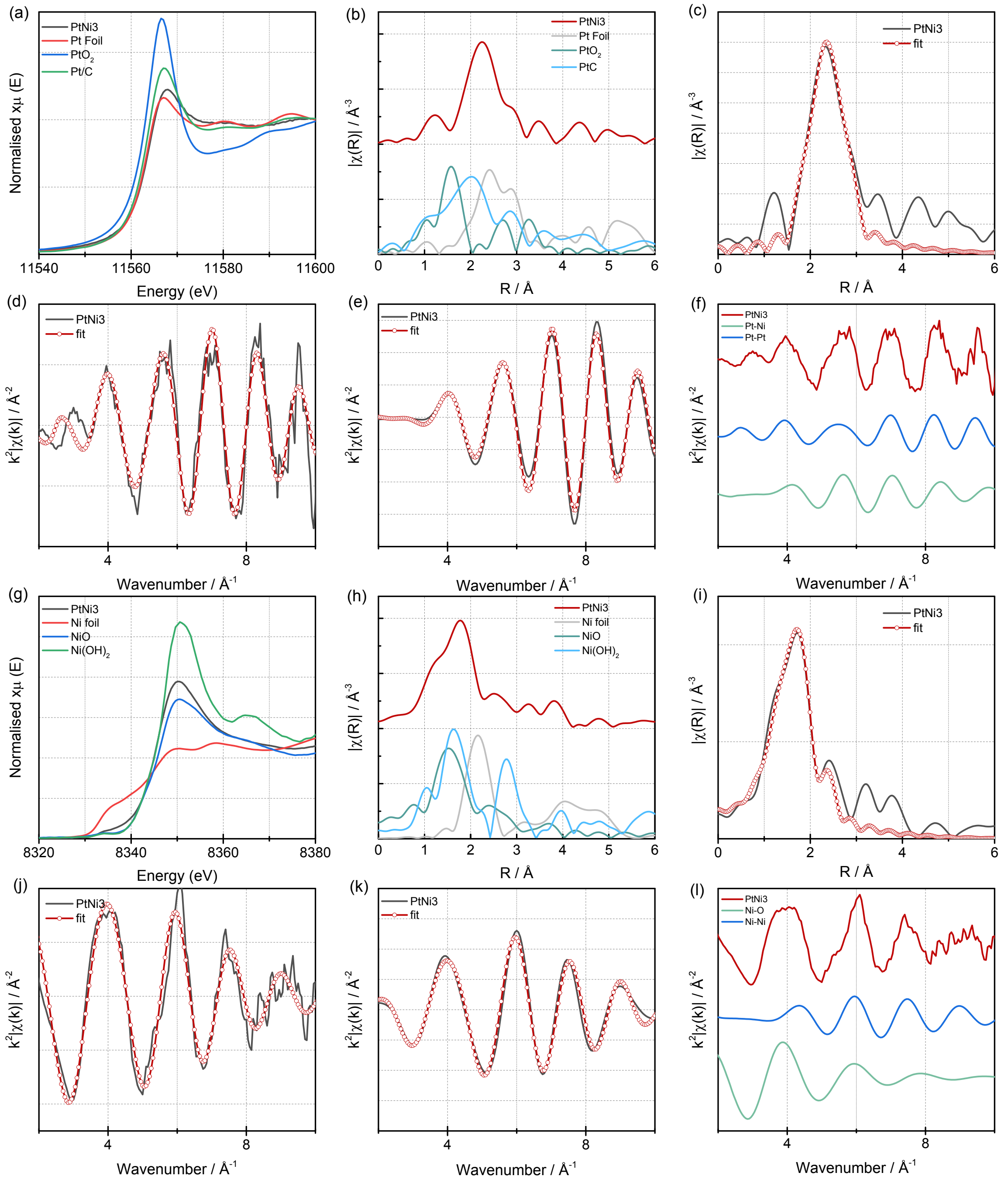

Figure S7 PtNi3 Pt L_3-edge: *a) XANES and b) EXAFS r-space, EXAFS fitting results in c) r-space, d) k-space, e) q-space and f) k-space contribution of each path.* ***PtNi3 Ni K-edge:*** *g) XANES and h) EXAFS r-space, EXAFS fitting results in i) r-space, j) k-space, k) q-space and l) k-space contribution of each path.*

Table S2 *EXAFS fitting results for three PtNi materials.*

sample	Edge	Scattering Pair	CN	R (Å)	ΔE_0 (eV)	σ^2 (10^{-3}Å^2)	S_0^2	R factor
PtNi1	Pt L_3	Pt-Pt	7.41	2.72	6.77 +/-1.67	13.00	0.714	1.97%
			+/- 2.66	+/-0.01		+/-5.21		
		Pt-Ni	4.29	2.64		5.87		

			+/-1.48	+/-0.01		+/-2.49		
	Ni K	Ni-Ni	1.40	2.54	-11.74 +/-5.33	2.29	0.697	0.62%
			+/-0.83	+/-0.01		+/-6.11		
		Ni-Pt	4.27	2.60		5.37		
			+/-2.27	+/-0.01		+/-5.31		
		Ni-O						
PtNi2	Pt L_3	Pt-Pt	6.29	2.73	3.03 +/-2.35	4.17	0.714	2.52%
			+/-2.91	+/-0.01		+/-3.04		
		Pt-Ni	6.67	2.61		19.5		
			+/-2.46	+/-0.03		+/-5.88		
	Ni K	Ni-Ni	1.30	2.61	10.15 +/-6.29	4.81	0.697	2.44%
			+/-0.94	+/-0.03		+/-2.31		
		Ni-Pt	3.48	2.74		13.81		
			+/-1.90	+/-0.03		+/-10.32		
		Ni-O	4.97	2.15		9.31		
			+/-2.89	+/-0.02		+/-8.6		
PtNi3	Pt L_3	Pt-Pt	5.76	2.72	2.97 +/-2.97	13.02	0.714	2.97%
			+/-3.45	+/-0.02		+/-1.33		
		Pt-Ni	5.20	2.58		5.48		
			+/-1.63	+/-0.03		+/-3.75		
	Ni K	Ni-Ni	4.17	2.43	1.76 +/-4.18	11.1	0.697	2.1%
			+/-3.95	+/-0.02		+/-9.42		
		Ni-Pt						
		Ni-O	6.47	2.08		16.98		
			+/-1.98	+/-0.05		+/-8.53		

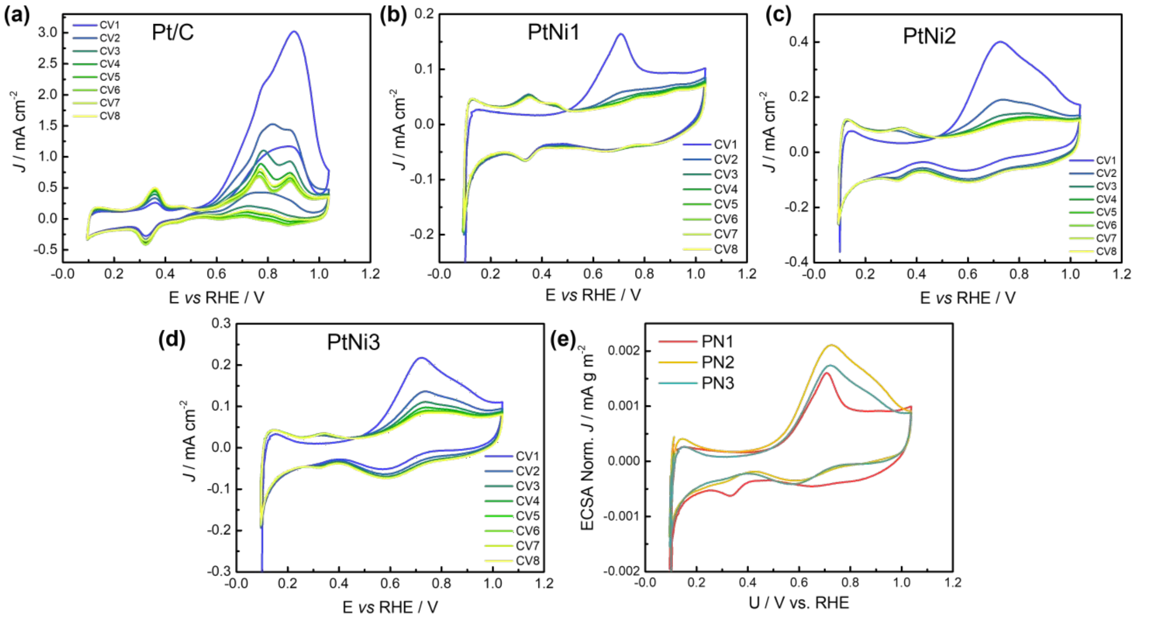

Figure S8 *(a-d) Glycerol stripping profiles of Pt/C and PtNi electrocatalysts. 8 scans are recorded for each measurement. (e) The first scan of glycerol stripping for three PtNi catalysts normalized by the corresponding ECSA values.*

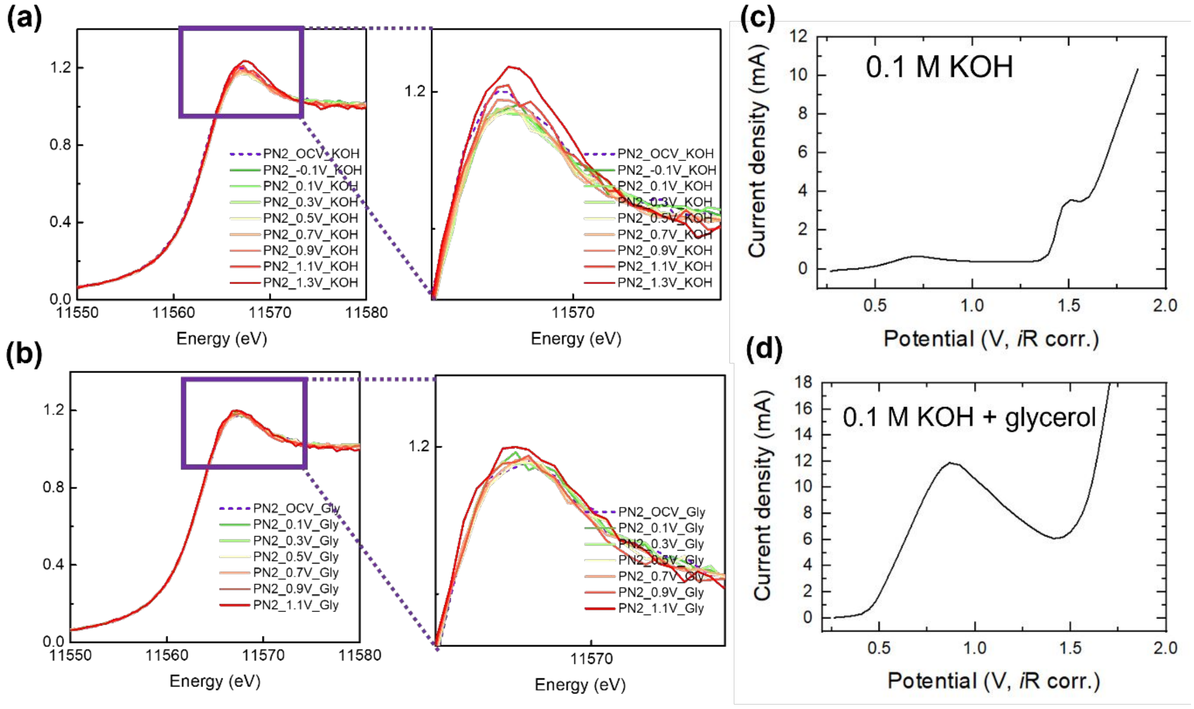

Figure S9. *operando XANES spectra at Pt L3-edge for PtNi2 in (a) 1 M KOH and (b) 1 M KOH/0.1 M glycerol; the recorded electrochemical data during operando optical-electrochemical experiments for PtNi2.*

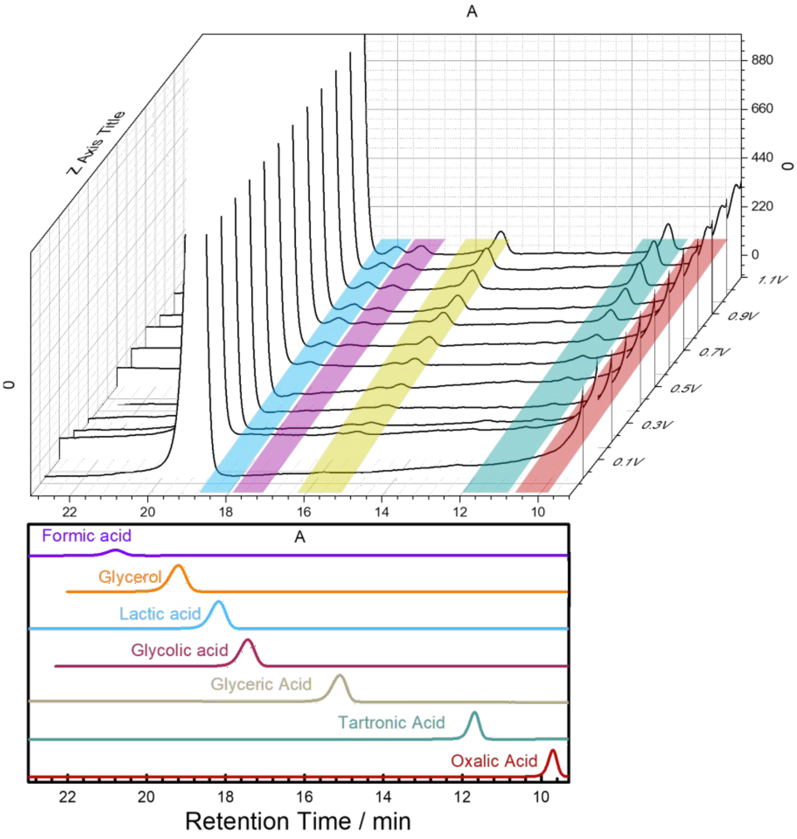

Figure S10 *HPLC signal of Pt/C online sampling at each potential. The colored regions are peaks associated to lactic acid (blue), glycolic acid (purple), glyceric acid (yellow), tartronic acid (cyan) and oxalic acid (red). The signals from the reference chemicals are also plotted here.*

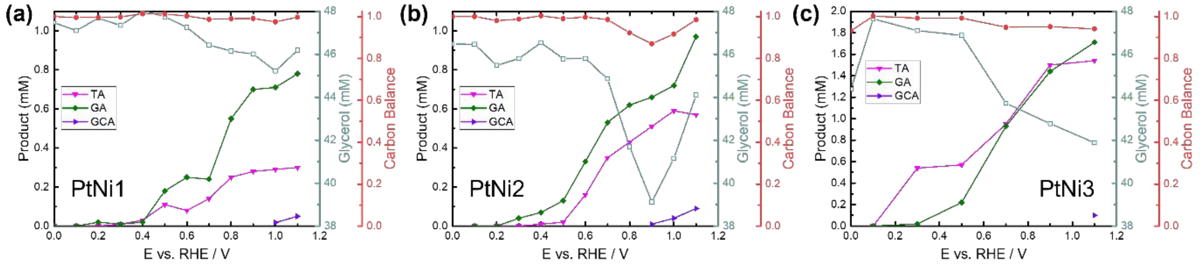

Figure S11 *Online sampling + HPLC for PtNi materials*

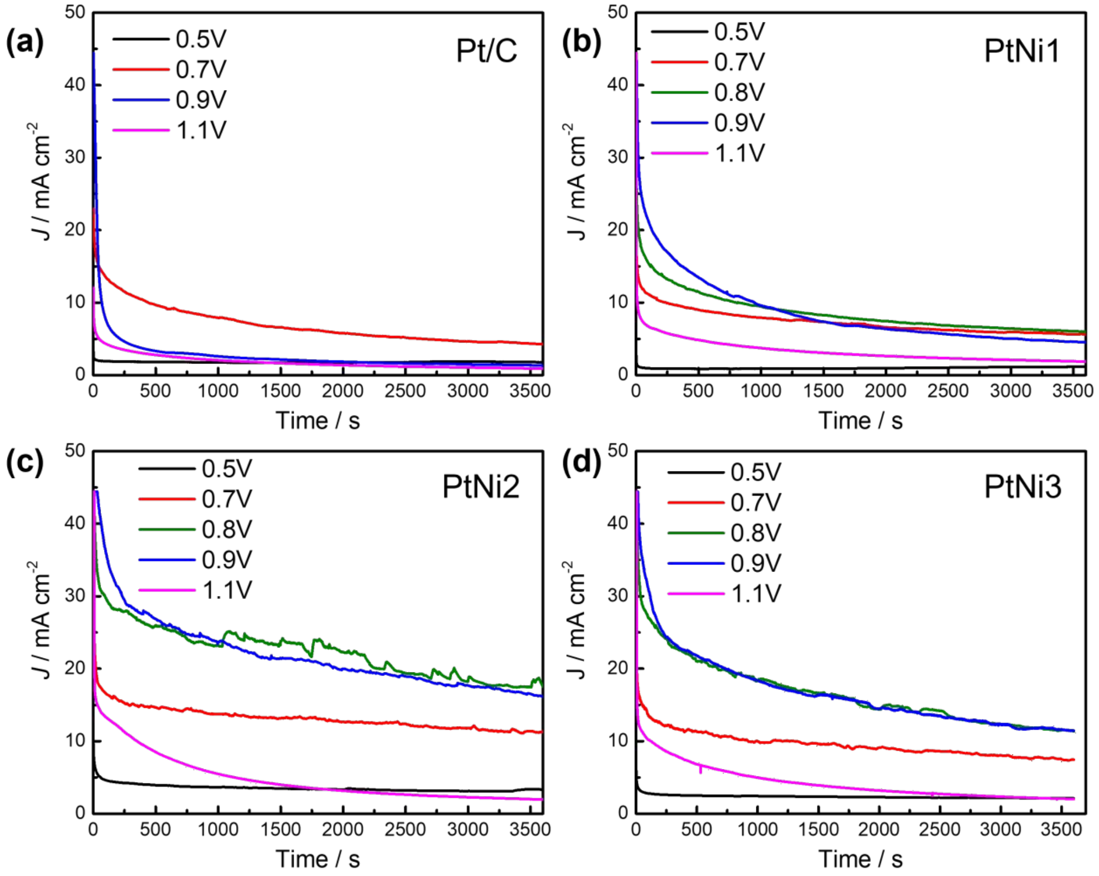

Figure S12 *1h chronoamperometry data for product accumulation.*

(1) Cui, C.; Gan, L.; Heggen, M.; Rudi, S.; Strasser, P. Compositional Segregation in Shaped Pt Alloy Nanoparticles and Their Structural Behaviour during Electrocatalysis. *Nat. Mater.* **2013**, *12* (8), 765–771.

(2) Rudi, S.; Cui, C.; Gan, L.; Strasser, P. Comparative Study of the Electrocatalytically Active Surface Areas (ECSAs) of Pt Alloy Nanoparticles Evaluated by Hupd and CO-Stripping Voltammetry. *Electrocatalysis* **2014**, *5* (4), 408–418.

(3) Garcia, A. C.; Kolb, M. J.; Van Nierop Y Sanchez, C.; Vos, J.; Birdja, Y. Y.; Kwon, Y.; Tremiliosi-Filho, G.; Koper, M. T. M. Strong Impact of Platinum Surface Structure on Primary and Secondary Alcohol Oxidation during Electro-Oxidation of Glycerol. *ACS Catal.* **2016**, *6* (7), 4491–4500.

(4) Bott-Neto, J. L.; Rodrigues, M. V. F.; Silva, M. C.; Carneiro-Neto, E. B.; Wosiak, G.; Mauricio, J. C.; Pereira, E. C.; Figueroa, S. J. A.; Fernández, P. S. Versatile Spectroelectrochemical Cell for In Situ Experiments: Development, Applications, and Electrochemical Behavior**. *ChemElectroChem* **2020**, *7* (21), 4306–4313.

(5) Zhang, Z.; Xin, L.; Li, W. Electrocatalytic Oxidation of Glycerol on Pt/C in Anion-Exchange Membrane Fuel Cell: Cogeneration of Electricity and Valuable Chemicals. *Appl. Catal. B Environ.* **2012**, *119–120*, 40–48.

(6) Nitopi, S.; Bertheussen, E.; Scott, S. B.; Liu, X.; Engstfeld, A. K.; Horch, S.; Seger, B.; Stephens, I. E. L.; Chan, K.; Hahn, C.; Nørskov, J. K.; Jaramillo, T. F.; Chorkendorff, I. Progress and Perspectives of Electrochemical CO2 Reduction on Copper in Aqueous Electrolyte. *Chem. Rev.* **2019**, *119* (12), 7610–7672.
